# Single-cell Raman spectroscopy identifies *Escherichia coli* persisters and reveals their enhanced metabolic activities

**DOI:** 10.3389/fmicb.2022.936726

**Published:** 2022-08-04

**Authors:** Chuan Wang, Rongze Chen, Jian Xu, Lijian Jin

**Affiliations:** ^1^Faculty of Dentistry, The University of Hong Kong, Hong Kong, Hong Kong SAR, China; ^2^Single-Cell Center, CAS Key Laboratory of Biofuels, Shandong Key Laboratory of Energy Genetics and Shandong Energy Institute, Qingdao Institute of Bioenergy and Bioprocess Technology, Chinese Academy of Sciences, Qingdao, China; ^3^College of Life Science, University of Chinese Academy of Sciences, Beijing, China

**Keywords:** single-cell Raman spectroscopy, *E. coli*, persister, metabolism, persister resuscitation

## Abstract

Microbial persisters are the featured tiny sub-population of microorganisms that are highly tolerant to multiple antimicrobials. Currently, studies on persisters remain a considerable challenge owing to technical limitations. Here, we explored the application of single-cell Raman spectroscopy (SCRS) in the investigation of persisters. *Escherichia coli* (ATCC 25922) cells were treated with a lethal dosage of ampicillin (100 μg/mL, 32 × MIC, 4 h) for the formation of persisters. The biochemical characters of *E. coli* and its persisters were assessed by SCRS, and their metabolic activities were labeled and measured with D_2_O-based single-cell Raman spectroscopy (D_2_O-Ramanometry). Notable differences in the intensity of Raman bands related to major cellular components and metabolites were observed between *E. coli* and its ampicillin-treated persisters. Based on their distinct Raman spectra, *E. coli* and its persister cells were classified into different projective zones through the principal component analysis and t-distributed stochastic neighbor embedding. According to the D_2_O absorption rate, *E. coli* persisters exhibited higher metabolic activities than those of untreated *E. coli*. Importantly, after the termination of ampicillin exposure, these persister cells showed a temporal pattern of D_2_O intake that was distinct from non-persister cells. To our knowledge, this is the first report on identifying *E. coli* persisters and assessing their metabolic activities through the integrated SCRS and D_2_O-Ramanometry approach. These novel findings enhance our understanding of the phenotypes and functionalities of microbial persister cells. Further investigations could be extended to other pathogens by disclosing microbial pathogenicity mechanisms for developing novel therapeutic strategies and approaches.

## Introduction

Microorganisms develop multiple strategies to overcome numerous stresses and harsh conditions for survival, such as oxidative and nitrosative stress (Brown et al., 2009), lack of nutrition (Gore and Payne, 2010), extreme pH microenvironments (Marcus et al., 2016) and/or temperature (Ochiai et al., 2020), and antimicrobial treatments (Grubor et al., 2006; Requena, 2012). Herein, the formation of persisters is considered to be one of the most important mechanisms. Microbial persisters refer to a tiny subset of microorganisms that are highly tolerant to multiple antimicrobials. Persister cells have no acquired genetic mutants and are non-inheritable, which is different from antibiotic-resistant cells (Lewis, 2010). When the majority of the microbial population is killed by a lethal concentration of antimicrobials, persisters remain to survive and yet resume growth after the termination of antimicrobial treatments, thereby forming a new population that is genetically identical to the original one (Balaban et al., 2019). As such, microbial persisters are regarded as the main reason for the relapse and recalcitrance of various chronic infections in humans (Lewis, 2010; Helaine and Kugelberg, 2014).

The concept of microbial persistence was first described by Bigger (1944). Since 2000, there are increasing scientific reports on the deleterious persisters of nearly all bacteria and fungi tested (Van Den Bergh et al., 2017). *Escherichia coli* is commonly used in microbiological research, and indeed many important findings on persisters arise from various experiments with *E. coli*. For instance, Balaban and colleagues used a microfluidic device to observe *E. coli* persisters at the single-cell level, and divided them into stationary phase-induced persisters (type I) and spontaneous persisters (type II) (Balaban et al., 2004, 2019; Kaldalu et al., 2016). However, further investigations on the profiles, metabolisms, and functionality of *E. coli* persisters remain stagnant, owing to various technical limitations, such as the lack of precise isolation and labeling approaches. The recent advance in biotechnology has witnessed a considerable number of integrative investigations on persisters with various approaches and new perspectives in different fields of biomedical sciences (Gollan et al., 2019). However, different notions and even conflicting opinions still exist about the identification, survival mechanisms, growth, and metabolic activities of persisters. Earlier studies show that persisters are generally present in a non- or slow-growing state, and dormancy is an important survival strategy (Bigger, 1944; Lennon and Jones, 2011; Harms et al., 2016). While recent research work tends to appreciate that persisters could actually divide and grow even under lethal dosages of antimicrobials (Orman and Brynildsen, 2013a; Wakamoto et al., 2013; Ueno et al., 2019), further investigations are therefore highly required to clarify these crucially important points. Moreover, traditional work on the whole population of persisters has well-documented their unique adaptive characteristics, through analyzing the biphasic kill curves and assessing the expression profiles of genes and proteins (Tian et al., 2019; Sulaiman and Lam, 2020; Yee et al., 2020). The latest definitions and guidelines for studies on persister cells emphasize the profound importance of targeting these phenotypic variants “originating from a population that displays antibiotic persistence” *via* a single-cell-based approach (Balaban et al., 2019). Thus, more advanced technologies would enable us to detect “what happened” inside every single cell of these noxious persisters, for further revealing the underlying survival mechanisms and pathogenicity as well as developing tackling strategies in the near future.

Single-cell Raman spectroscopy (SCRS) is a non-intrusive technology to identify the biochemical characteristics of a single cell, by employing a homochromous laser as a light source to obtain cellular Raman spectra. Here, these specific spectra, like the molecular “fingerprint” of an individual cell, provide comprehensive and intrinsic profiles of the cell (Huang et al., 2010; Smith et al., 2016). Recently, SCRS has been utilized to categorize different types of bacterial cells (Harz et al., 2009; Stockel et al., 2012), measure metabolic products (Ji et al., 2014; He et al., 2017), check metabolic states (Athamneh et al., 2014; Teng et al., 2016), rapidly sort and isolate single cells (Zhang et al., 2015; Wang et al., 2017), and identify antibiotic resistance (Cui et al., 2016; Germond et al., 2018; Verma et al., 2021). As such, SCRS may facilitate novel studies on precisely defining persisters and monitoring their biochemical dynamics.

Generally, metabolism refers to the biochemical reactions that are processed within an individual viable cell, for living and reproduction, through obtaining essential energy and nutrients. It does play a crucial role in the formation of microbial persisters, and therefore, targeting the key metabolic pathways could be a promising strategy for tackling persister cells (Amato et al., 2014). Traditionally, measurement of metabolic levels focuses on a single pathway like respiratory activity (Huang et al., 2019), or complicated metabonomic–proteomic techniques are performed to evaluate the total metabolites of the whole microbial population (Li et al., 2018). It is worthy to note that newly developed approaches enable us to track the fate of essential chemical elements, such as hydrogen, carbon, and nitrogen, using their isotopes (Wang et al., 2016; Nikolic et al., 2017; McClelland et al., 2020). As H_2_O is an essential element of metabolism in living cells, tracking and quantifying the transition from H to D are excellent alternatives to assess the metabolic activities of the targeting microbes and their persisters (Tao et al., 2017).

Following the treatment of heavy water (D_2_O), the H from H_2_O is replaced by D from D_2_O during various cellular biosynthesis processes. Notably, the D is combined with C to form the carbon-deuterium Raman band (C-D band), which can be detected constantly by Raman spectroscopy, and the amount of deuterium absorbed by a single cell could be quantitated according to the switch from the C-H band to C-D band (Berry et al., 2015). Therefore, the D_2_O-based SCRS (D_2_O-Ramanometry) can function as a non-invasive, quantifiable, and valuable approach to analyzing the metabolic activity of cells under different conditions (Wang et al., 2016; Tao et al., 2017). Indeed, novel D_2_O-Ramanometry-based methods for rapid tests of antimicrobial and anticancer effects have been recently developed by others and our group (Tao et al., 2017; Yang et al., 2019; Hekmatara et al., 2021). This technology can be favorably extended to investigate the metabolism of persisters in an advanced manner. For instance, Ueno and colleagues have analyzed the metabolic activities of *Mycobacterium tuberculosis* through the combined SCRS and D_2_O labeling, and revealed that persisters were not in a dormant state, while their resuscitation-related metabolic activities were not analyzed (Ueno et al., 2019).

In the present study, the Raman spectra of *E. coli* persisters at different growth stages were identified by SCRS, and the metabolic activities during the formation and resuscitation of persister cells were analyzed with D_2_O-Ramanometry. This work enriches our knowledge of the featured phenotypes and unexpected functionalities of microbial persister cells, and may contribute to advancing the basic and translational research in this critical field of microbiology.

## Materials and methods

### Bacterial culture and persister formation

*E. coli* (ATCC 25922) was used and cultured as previously described (Duan et al., 2016). Briefly, bacteria maintained as frozen stock (30% glycerol + 70% bacteria, stored in a −80°C freezer) were first grown on Luria-Bertani (LB) broth agar plates (10 g/L NaCl, 10 g/L tryptone, and 5 g/L yeast extract with 15 g/L agar) overnight in an aerobic atmosphere at 37°C. A single colony was picked into 5 mL of liquid LB broth (without agar) in 15-mL universal tubes and cultured with shaking at 200 rpm for 12 h under the same conditions.

According to our preliminary result, the MIC of ampicillin on the *E. coli* strain used in the present study (ATCC 25922) is 3.125 μg/mL. Persister formation was conducted as previously described (Kaldalu et al., 2016). In brief, overnight planktonic-cultured *E. coli* (12 h) was 1:100 diluted into 20 mL fresh LB broth in 50-mL universal tubes and further cultured at 37°C with shaking at 200 rpm for different time points (1, 2, 3, 4, 6, and 12 h), followed by ampicillin treatment (100 μg/mL, 32 × MIC) for 4 h. To determine the number of viable persister cells, 100-μL aliquots were washed two times with PBS and subjected to 10-fold serial dilution in PBS, followed by plate culture using Autoplate^®^ Spiral Plating System (Advanced Instruments, Norwood, MA) to test the bacterial viability based on the colony-forming unit (CFU) counts.

### Growth curve of *E. coli*

The growth curve of *E. coli* was constructed by photometric measurement and CFU counts, respectively. In short, overnight planktonic-cultured *E. coli* (12 h) was 1:100 diluted into 20 mL of fresh LB broth in 50-mL universal tubes (11 tubes in total), and then cultured at 37°C with shaking at 200 rpm. At different time points (0, 1, 2, 3, 4, 5, 6, 7, 8, 10, and 12 h), 1 mL aliquots were taken from one tube and OD_600_ was recorded using SpectraMax^®^ M2 Multimode Microplate Reader (Molecular Devices Ltd., Sunnyvale, CA). The same volume (1 mL) of fresh LB broth was used for the zero setting. At the same time, 100-μL aliquots were taken from the same tube and subjected to 10-fold serial dilution in PBS, followed by plate culture to determine the bacterial viability based on the CFU counts.

### Cell growth observation using agarose pad

Agarose pad was prepared as previously described (Howell et al., 2017). A square was cut out from the center of a laboratory film (22 mm × 22 mm) leaving a ~2–5 mm border to serve as a gasket for the agarose pad. The gasket was then placed onto a sterilized glass slide (75 mm × 25 mm) and heated until the film slightly melted onto the glass. Agarose solution (0.3 g of agarose with 20 mL of fresh LB) was heated in a microwave until the solution became clear. Around 70 μL of agarose solution was pipetted into the center of the gasket and covered with a coverslip to evenly distribute the agarose. After solidification, the coverslip was removed from the agarose pad and a small strip of agarose was removed to create an air pocket. *E. coli* persister cells were isolated by treatment with ampicillin (100 μg/mL) for 4 h and resuspended in fresh LB broth. For the observation of persister growth, ampicillin at 100 μg/mL was maintained in LB broth and added to the agarose pad. For the observation of persister resuscitation, persister cells were washed two times with PBS and diluted into fresh LB broth without ampicillin. About 1 μL of persister cells after resuspension were spotted on the agarose pad, followed by coverage with a new coverslip. Edges of the coverslip were sealed with petroleum jelly. The samples were then observed by an Eclipse E200 optical microscope (Nikon, Tokyo, Japan) using × 100 oil immersion objectives. Series photos were manually taken every 10 min until 2–3 h, followed by analysis using NIS-Elements Viewer (Nikon, Tokyo, Japan).

### Single-cell Raman spectrometry and machine learning modeling

The sample was prepared as previously described (Yang et al., 2019). Briefly, bacteria were collected by centrifuging at 8,000 g for 1 min and then washed two times using Milli-Q water. An aliquot of the sample (1.5 μl) was spotted on CaF_2_ slides with a low background signal and dehydrated in the air at room temperature. Single-cell Raman spectra were acquired using a WiRE5.3 (Renishaw) confocal micro-Raman system armed by an excitation laser (532 nm Nd: YAG) and a diffraction grating (900 grooves/mm). A dry objective (100×) with a numerical aperture of 0.9 (Olympus, Japan) was used for the observation of bacterial cells and Raman signal acquisition. The laser power on the sample plane was ~30 mW/μm^2^. Raman spectra were acquired in the range of 400–3,200 cm^−1^ with a spectral resolution of ~2 cm^−1^. The acquisition time for the individual spectrum was 5 s, and at least 50 valid single-cell spectra were attained from each sample following quality control. The principle of quality control is removing spectra that represent the background signal, impurity signal, and those with obvious fluorescence signal. After preprocessing Raman spectra (500–1,750 cm^−1^) *via* baseline correction and normalization with the sum of intensity, both principal component analysis (PCA) and t-distributed stochastic neighbor embedding (t-SNE) methods were used for visualization of data structure. After comparing various commonly used classifiers, partial least squares discrimination analysis (PLS-DA) and support vector machine (SVM) were used for modeling due to their high accuracy and operation efficiency, and the data from different groups were then randomly distributed into training and test sets (7:3). The number of components included in the PLS-DA model was 20, and the Mahalanobis Distance was employed as discriminant methods to foresee the class of new data. Radial basis, as the kernel function, was employed in training and for predicting the SVM model.

### D_2_O labeling and calculation of C-D ratio

For D_2_O labeling, liquid LB broth was prepared with different percentages (v/v) of D_2_O (99.8 atom % D, Aldrich, Milwaukee, WI) and then sterilized by passing through a 0.22-μm filter without autoclaving. *E. coli* and *E. coli* persisters were washed two times with Milli-Q water and cultured in LB broth with different percentages of D_2_O for various time periods prior to acquiring single-cell Raman spectra. After preprocessing Raman spectra (1,750–3,200 cm^−1^) *via* baseline correction and normalization with max intensity, batch calculations of the C-D ratio [CD/(CD + CH)] from at least 50 single-cell Raman spectra were undertaken within each group. C-D ratio, reflecting the metabolic activity of a single cell, was calculated by dividing the C-D band area (2,050–2,300 cm^−1^) by the sum of the C-D and C-H band areas (2,800–3,050 cm^−1^).

### Resuscitation of *E. coli* persisters

Overnight planktonic-cultured *E. coli* (12 h) was 1:100 diluted into 20 mL of fresh LB broth in 50-mL universal tubes and further cultured at 37°C with shaking at 200 rpm for 3 and 6 h, followed by ampicillin treatment (100 μg/mL, 32 × MIC) for 4 h. Persister cells after ampicillin treatment were washed two times with Milli-Q water and further cultured in 20 mL of fresh LB broth without the drug in 50- mL universal tubes for another 4 h under the same conditions. Single-cell Raman spectra were obtained afterward as described above. The metabolic activities of *E. coli* persisters during resuscitation were assessed by observing their D_2_O absorption rate. Briefly, persister cells after ampicillin treatment (100 μg/mL, 4 h) were washed two times and cultured in 100% D_2_O medium without drugs for another 4 h, and single-cell Raman spectra were acquired every 1 h. The C-D ratio was calculated as mentioned above.

### Statistical analysis

At least three independent repeats were conducted separately for each experiment. Data were presented as one randomly chosen sample from the three independent experiments. All statistical analysis was made using R (Version 4.0) by customized scripts. The relevant figures were plotted *via* the ggplot 2 package. The inter-group difference was examined using the student's *t*-test *via* package “ggpubr”. Statistical significance was determined with a *P*-value < 0.05 with a 95% confidence interval. When detecting the statistical significance level between different spectra, machine learning was adopted *via* the package of “mixOmics”, “Rtsne”, “pls”, “e1071”, “Matrix”, “kknn” and “randomForest”.

## Results

### Single-cell Raman spectra of *E. coli* persisters

**Figure 1:**
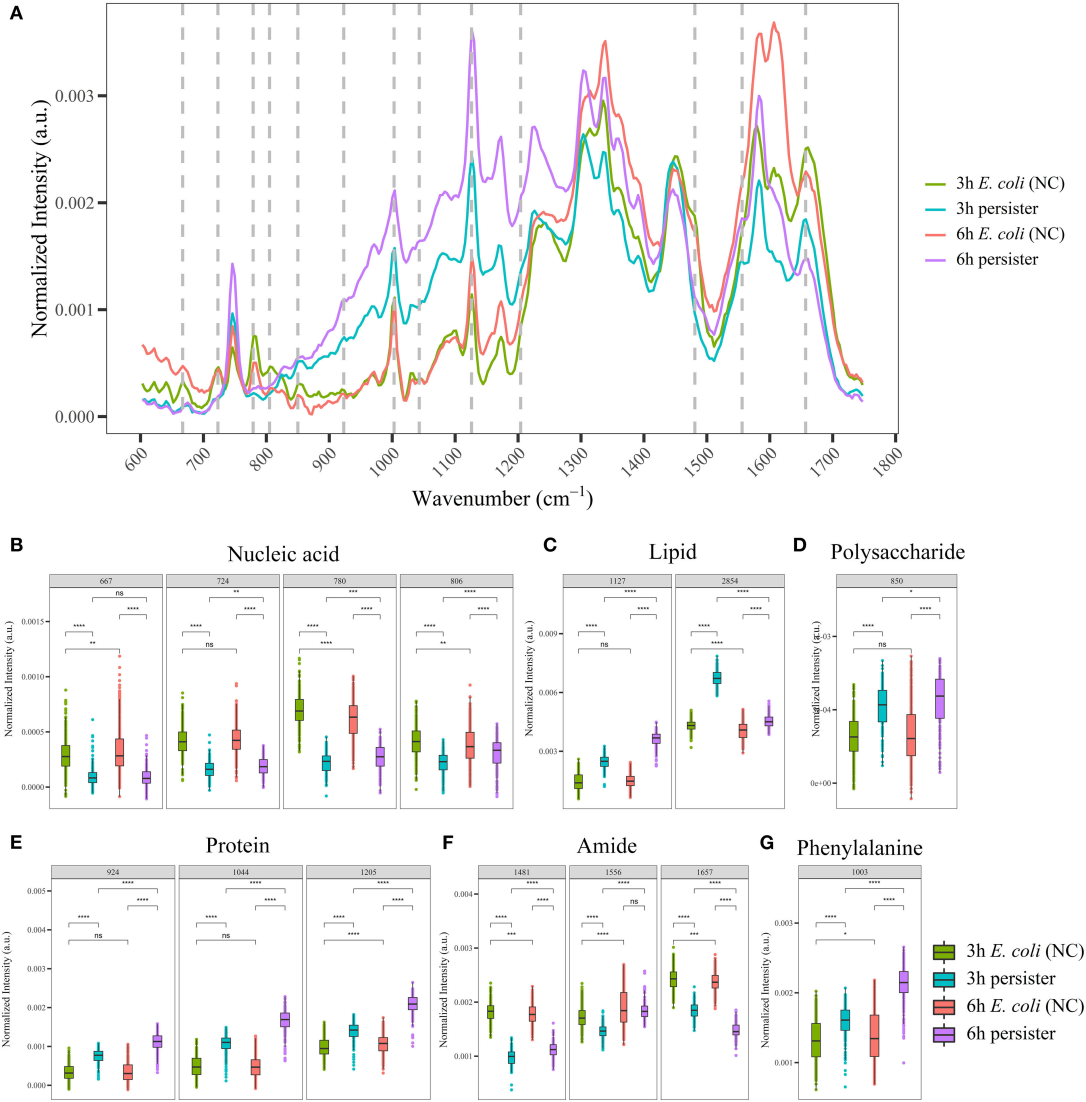
Single-cell Raman spectra of *E. coli* persisters. Overnight planktonic-cultured *E. coli* (12 h) was 1:100 diluted into fresh LB broth and further cultured for 3 and 6 h, followed by ampicillin treatment (100 μg/mL, 32 × MIC) for 4 h. Single-cell Raman spectra were acquired afterward. **(A)** Mean spectra of *E. coli* and its persisters, normalized by the sum of fingerprint area from 600 to 1,750 cm^−1^. **(B–G)** Intensity of Raman bands for major cellular components: nucleic acid **(B)**, lipid **(C)**, polysaccharide **(D)**, proteins **(E)**, amide **(F)**, and phenylalanine **(G)**. ns, no significance; NC, *E. coli* cells without ampicillin treatment; *P < 0.05; **P < 0.01; ***P < 0.001, ****P < 0.0001.

The growth curve of *E. coli* showed that it reached the exponential phase at 3 h and the early stationary phase at 6 h from the initial culture (Supplementary Figure 1). *E. coli* persisters were produced as previously described (Kaldalu et al., 2016). After ampicillin treatment at 100 μg/mL for 4 h, the kill curves exhibited a biphasic pattern (Supplementary Figure 2), and the remaining cells showed neither growth nor lysis (Supplementary Figure 3) but were able to resuscitate after the termination of antibiotic exposure (Supplementary Figure 4), suggesting that they were the survival persister cells. The single-cell Raman spectra of *E. coli* and its persister cells obtained from different time points (1, 2, 3, 4, 6, and 12 h) were recorded and analyzed (Figure 1A, Supplementary Figure 5).

For single-cell Raman spectra of *E. coli* and its persisters at different states, notable changes in the intensity of Raman bands related to major cellular components and metabolites were observed (Supplementary Table 1), including nucleic acid, lipids, polysaccharides, proteins, amide, and phenylalanine (Figures 1B–G). The intensity of nucleic acid-related bands was significantly lower in the persister cells at both 3 and 6 h when compared to the counterpart *E. coli* cells (Figure 1B), whereas the intensities of lipid- and polysaccharide-related bands were significantly higher in the persister cells with reference to *E. coli* cells (Figures 1C,D). Various protein-related bands, such as at 924, 1,044, 1,205, and 1,003 cm^−1^ (phenylalanine), presented with enhanced intensities (Figures 1E,G), while amide-related bands had reduced intensity in the persister cells (Figure 1F).

The single-cell Raman spectra of *E. coli* and its persisters after 24 h of treatment were recorded and analyzed as well (Supplementary Figure 6). Here, the inter-group differences in the Raman bands related to these major cellular components/metabolites became less obvious with reference to the 4-h culture results, suggesting both *E. coli* and persister cells may enter the decline phase due to the exhaustion of nutrients.

**Figure 2:**
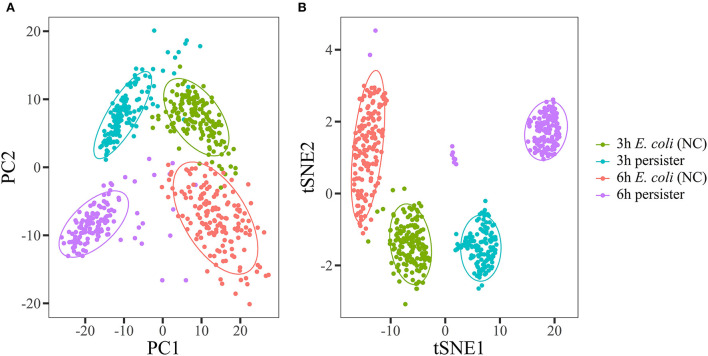
Raman spectra of *E. coli* and its persisters displayed with unsupervised tagging machine learning methods. Overnight planktonic-cultured *E. coli* (12 h) was 1:100 diluted into fresh LB broth and further cultured for 3 and 6 h, followed by ampicillin treatment (100 μg/mL, 32 × MIC) for 4 h. Single-cell Raman spectra were acquired afterward. Principal component analysis **(A)** and t-distributed stochastic neighbor embedding **(B)** were used to visually display the data structure of Raman spectra. *E. coli* and its persister cells were classified into different projective zones using both methods. *N* > 50 for each group. NC: *E. coli* cells without ampicillin treatment.

Principal component analysis (PCA) and t-distributed stochastic neighbor embedding (t-SNE) were used to visually demonstrate the data structure of Raman spectra. *E. coli* and its persister cells were classified into different projective zones (Figures 2A,B). Partial least squares discrimination analysis (PLS-DA) and support vector machine (SVM) methods were employed for modeling, and the data from different groups were randomly divided into training and test sets (7:3). The accuracy rates of both models (PLS-DA and SVM) were over 95% (Supplementary Tables 2A,B).

Taken together, these observations revealed that there was a considerable difference in single-cell Raman spectra between *E. coli* and its persisters.

### Dose- and time-dependent effects of D_2_O on the Raman spectra of *E. coli*

**Figure 3:**
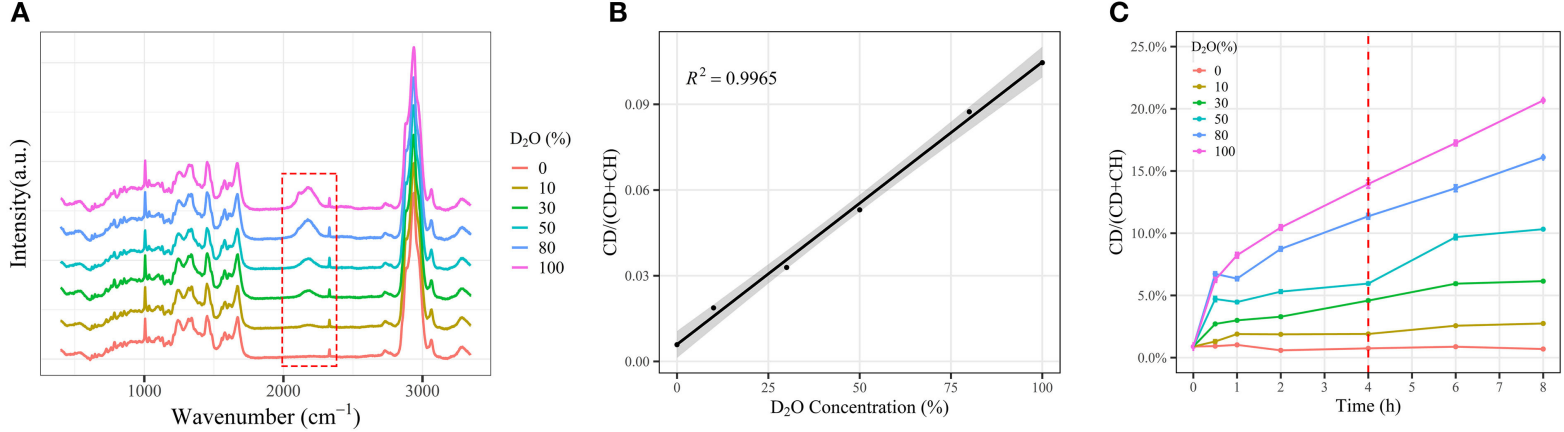
Effect of deuteroxide on cellular deuterium incorporation measured by Raman spectroscopy. *E. coli* cells were washed two times with Milli-Q water and cultured in LB broth with different percentages of D_2_O for various time periods prior to acquiring single-cell Raman spectra. **(A)** Single-cell Raman spectra of *E. coli* grown to stationary phase in LB media amended with 0, 10, 30, 50, and 100% D_2_O (*n* > 50). **(B)** Regression lines of C-D ratios from *E. coli* cells with respect to D_2_O percentages (*n* > 50). Mean C-D ratios (points) and 95% confidence intervals (light shade) are depicted. **(C)** Time- and dose-dependent CD ratios of *E. coli* in response to different percentages of D_2_O, respectively. Mean (solid lines) and standard error (shaded regions) from 50 individual cells are depicted with a 95% confidence interval.

The *E. coli* cells were cultured in LB broth containing different concentrations of D_2_O for 4 h. A broad peak occurred between 2,050 and 2,300 cm^−1^ in Raman spectra, corresponding to the formation of the C-D bond, and the intensity of the C-D peak increases in parallel with D_2_O concentration. No significant C-D peaks were found, when the cells were cultured in a D_2_O-free medium (Figure 3A). For the assessment of metabolic activity, the D incorporation rate [CDR, CD/(CD +CH)], was used to quantitatively represent the metabolic activity. It is noted that CDR had a linear correlation with the concentration of D_2_O through the linear fitting of CDR (Figure 3B). Besides, the CDR strength of the single cells of *E. coli* showed a time-dependent pattern as well (Figure 3C). Hence, D_2_O-Ramanometry may be a promising approach to evaluate the metabolic activities of a single cell.

### Metabolic activities of *E. coli* cells and its persister cells

**Figure 4:**
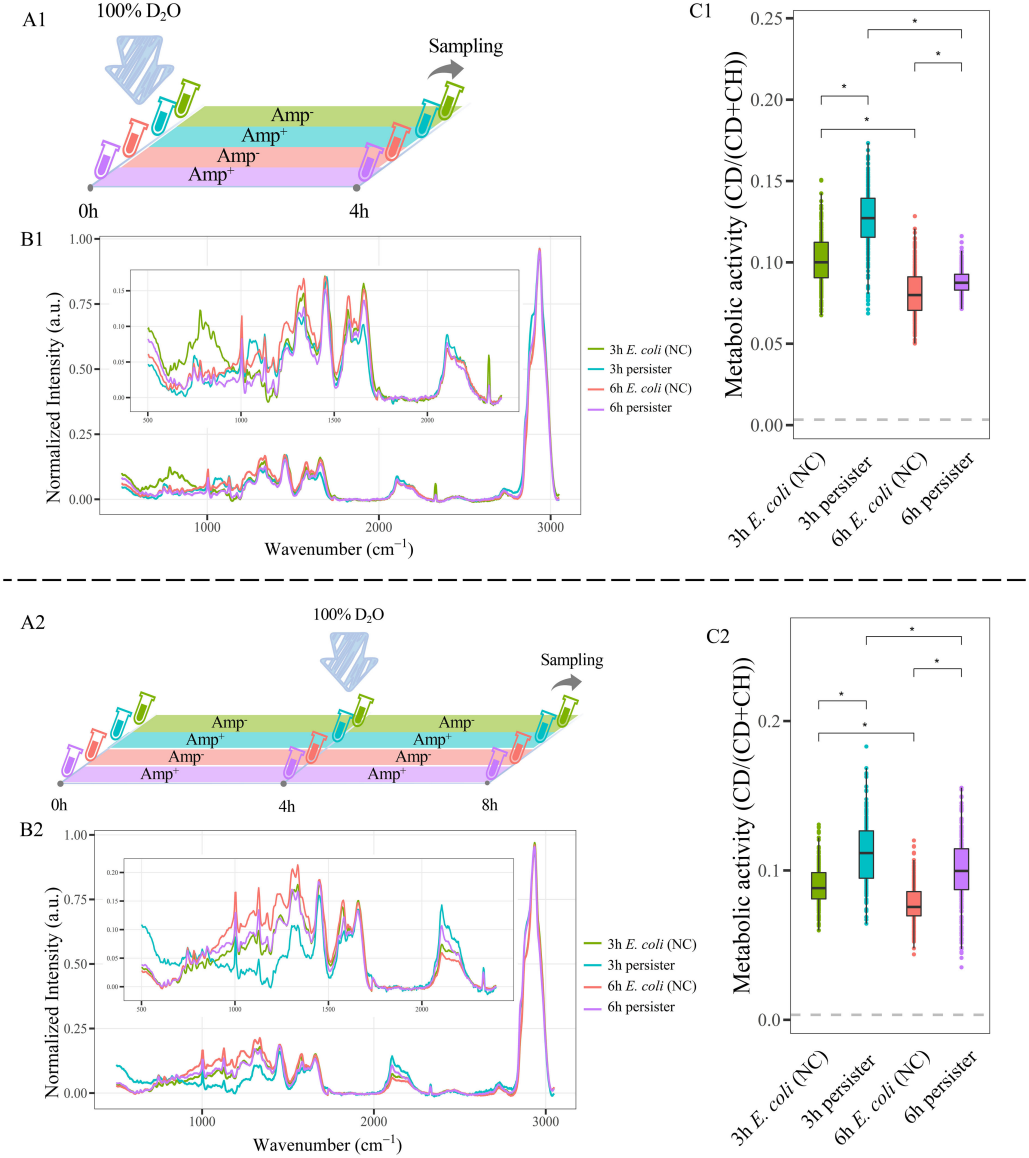
C-D ratio of *E. coli* cells and their persisters. **(A)** Workflow of D_2_O labeling strategy. **(A1)**: *E. coli* cells at 3 h and 6 h were cultured in LB broth with 100% D_2_O and ampicillin at 100 μg/mL for 4 h. **(A2)**: *E. coli* cells obtained at 3 and 6 h were treated with ampicillin (100 μg/mL, 4 h) for persister formation, followed by culture in LB broth with 100% D_2_O for another 4 h **(B1,B2)**: The average Raman spectra of 3 and 6-h persisters and their counterpart untreated *E. coli* cells, normalized by the max of CH peak from 2,800 to 3,050 cm^−1^. **(C1,C2)**: C-D ratio of persisters and *E. coli* cells. *N* > 50 for each group. NC: *E. coli* cells without ampicillin treatment; *P < 0.0001.

The metabolic activities of *E. coli* and its persisters were detected by D_2_O with different protocols. *E. coli* cells at 3 h and 6 h were cultured in LB broth with 100% of D_2_O and ampicillin at 100 μg/mL for 4 h (Figure 4A1). Alternatively, *E. coli* cells at 3 and 6 h were treated with ampicillin (100 μg/mL, 4 h) for persister formation, followed by culture in LB broth with 100% D_2_O for another 4 h (Figure 4A2). Raman spectra were acquired and analyzed afterward.

A significant difference was found between *E. coli* and its persisters in the Raman spectra (Figure 4B, Supplementary Figure 7). Importantly, the CDR of persister cells was higher than that of *E. coli* cells (Figure 4C). Since CDR represents the total metabolic activity within the targeting cell, the finding reflects that although persister cells are in a non- or slow-growing state (Supplementary Figure 3), furious metabolic activities are happening inside them.

### Single-cell Raman spectra of *E. coli* persisters during resuscitation

**Figure 5:**
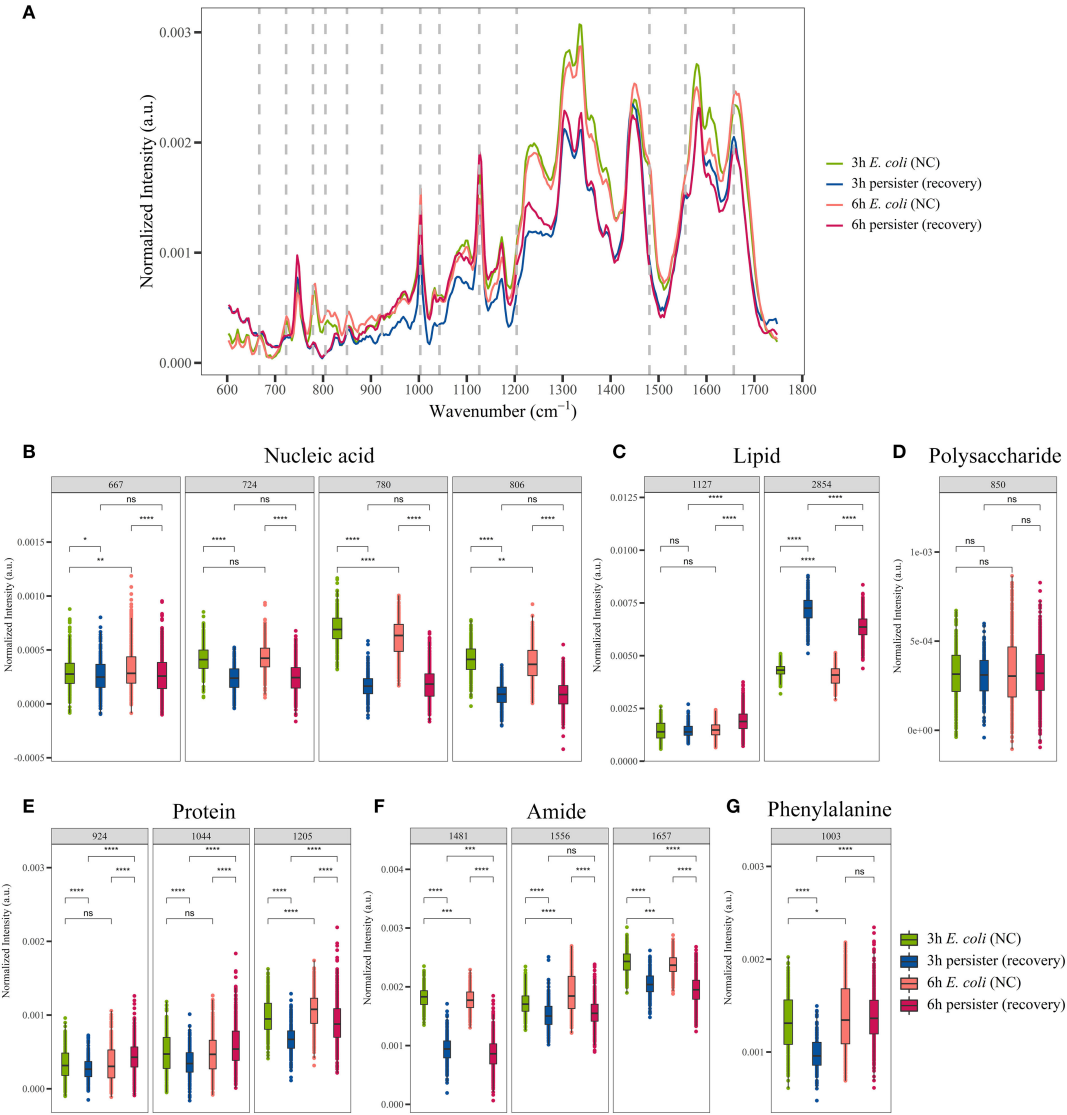
Single-cell Raman spectra of *E. coli* persisters during resuscitation. Overnight planktonic-cultured *E. coli* (12 h) was 1:100 diluted into fresh LB broth and further cultured for 3 and 6 h, followed by ampicillin treatment (100 μg/mL, 32 × MIC) for 4 h. Then, the persisters were washed two times with Milli-Q water and further cultured in fresh LB broth without the drug for another 4 h. Single-cell Raman spectra were obtained afterward. **(A)** Mean spectra of *E. coli* and persisters, normalized by the sum of fingerprint area from 600 to 1,750 cm^−1^. **(B–G)** Intensity of Raman bands for major cellular components: nucleic acid **(B)**, lipid **(C)**, polysaccharide **(D)**, proteins **(E)**, amide **(F)**, and phenylalanine **(G)**. *N* > 50 for each group. ns, no significance; NC, *E. coli* cells without ampicillin treatment; *P < 0.05; **P < 0.01; ***P < 0.001, ****P < 0.0001.

Of note, persister cells were able to resuscitate after the termination of antibiotic treatment (Supplementary Figure 4). Single-cell Raman spectra of *E. coli* and its persister cells after 4-h resuscitation were recorded and analyzed (Figure 5A, Supplementary Figure 8). Marked changes in Raman bands related to nucleic acid, lipids, polysaccharides, proteins, amide, and phenylalanine were detected (Figures 5B–G). After ceasing ampicillin treatment for 4 h, the intensities of nucleic acid- and amide-related bands in the persister cells remained at a lower level when compared to their counterparts (Figures 5B,F). The polysaccharide-related band had recovered, and there was no significant difference with the untreated *E. coli* cells (Figure 5D). The intensities of protein- and lipid-related bands varied, since some of them recovered while others did not (Figures 5C,E).

**Figure 6:**
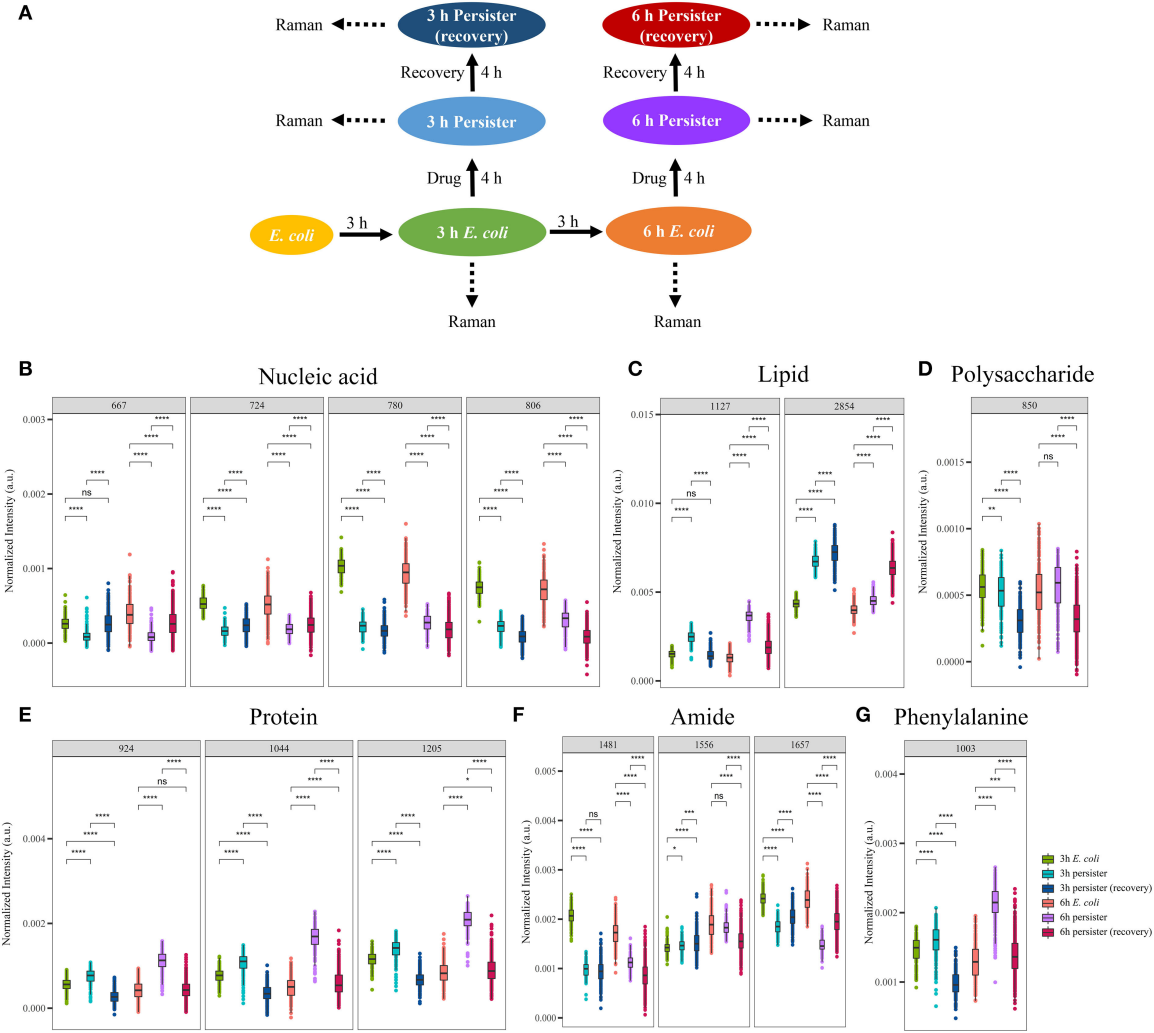
Single-cell Raman spectra of *E. coli* persisters before/after ampicillin treatment and after resuscitation. Overnight planktonic-cultured *E. coli* (12 h) was 1:100 diluted into fresh LB broth and further cultured for 3 and 6 h, followed by ampicillin treatment (100 μg/mL, 32 × MIC) for 4 h. Then, the persisters were washed two times with Milli-Q water and further cultured in fresh LB broth without the drug for another 4 h. Single-cell Raman spectra were acquired at different time points. **(A)** Workflow of Raman spectra detection. **(B–G)** Intensity of Raman bands for major cellular components: nucleic acid **(B)**, lipid **(C)**, polysaccharide **(D)**, proteins **(E)**, amide **(F)**, and phenylalanine **(G)**. *N* > 50 for each group. ns, no significance; * *P* < 0.05; ** *P* < 0.01; ***P < 0.001, **** *P* < 0.0001.

The Raman spectra of *E. coli* cells before and after ampicillin treatment, as well as following resuscitation, were analyzed for comparing the changes in the major cellular components and metabolites during the whole process (Figure 6A). Notably, the intensity of nucleic acid-related bands decreased sharply after ampicillin treatment (Figure 6B), while lipid-, polysaccharide-, protein-, amide-, and phenylalanine-related bands exhibited no obvious decrease or even slight increase for some compounds (Figures 6C–G). After the resuscitation, most of the bands showed a recovery trend (Figures 6B,E,G). While some bands, such as 2,854 cm^−1^ (related to acyl chains), showed a continuous increase (Figure 6C), others like 850 cm^−1^ (related to polysaccharide) and 1,481 cm^−1^/1,556 cm^−1^ (related to amide II) showed a continuous decrease (Figures 6D,F). Overall, Raman spectra of *E. coli* persister cells could, to a great extent, recover following the cessation of ampicillin treatment.

### Metabolic activity of *E. coli* persisters during the resuscitation

**Figure 7:**
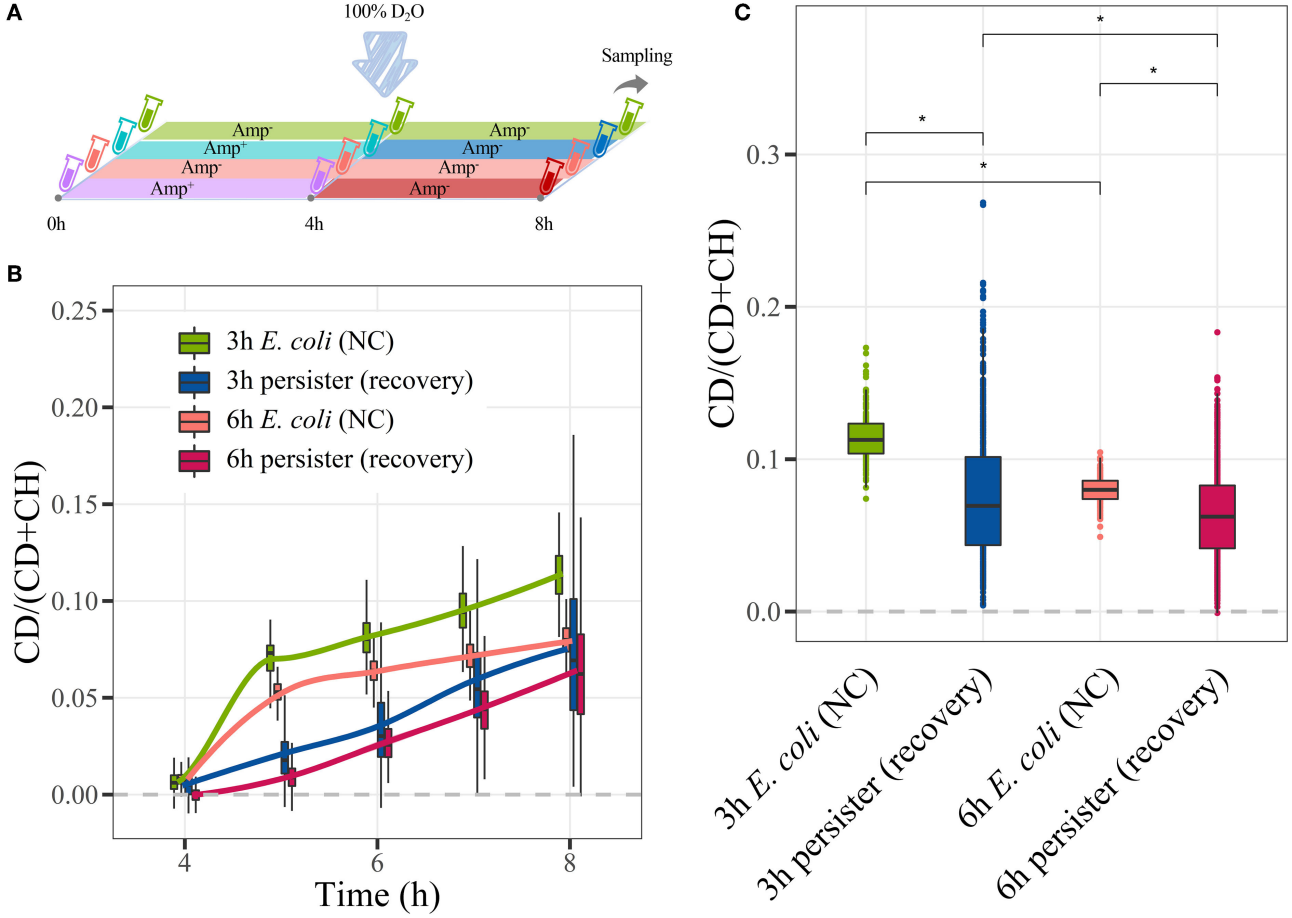
C-D ratio of *E. coli* persisters during resuscitation. Overnight planktonic-cultured *E. coli* (12 h) was 1:100 diluted into fresh LB broth and further cultured for 3 h and 6 h, followed by ampicillin treatment (100 μg/mL, 32 × MIC) for 4 h. Then, the persisters were washed two times with Milli-Q water and further cultured in LB broth with 100% D_2_O without the drug for another 4 h. Single-cell Raman spectra were obtained at every hour. **(A)** Workflow of D_2_O labeling strategy. **(B)** Time-dependent C-D ratio of persisters and *E. coli* cells during resuscitation. **(C)** C-D ratio of persisters and *E. coli* cells after 4 h of resuscitation. *N* > 50 for each group. NC: *E. coli* cells without ampicillin treatment; *P < 0.0001 .

The metabolic activities of *E. coli* persisters during resuscitation were assessed (Figure 7A). During the resuscitation of persisters, their CDR was consistently lower than that of *E. coli* cells (Figure 7B, Supplementary Figures 9A,B). Notably, persisters began to absorb D_2_O within 1 h after the termination of antibiotic treatment (Figure 7B). Furthermore, *E. coli* cells absorbed D_2_O in a biphasic pattern. For instance, the CDR of *E. coli* changed remarkably during the first hour of resuscitation and turned to a low yet stable trend for the following 3 h. In contrast, *E. coli* persisters maintained a persistent and stabilized CDR change rate during the 4-h observation period (Figure 7B). Indeed, the accumulated D_2_O level in the persister cells was lower than that observed in *E. coli* cells after 4 h (Figure 7C). These findings collectively showed that there was a certain difference in the metabolism of *E. coli* persisters during their formation and resuscitation, referring to *E. coli* cells.

## Discussion

Scientific studies on persisters have experienced a speedy expansion during the last two decades with the introduction of a variety of novel concepts and investigative approaches. However, conflict opinions remain on whether persisters are exactly under a dormant condition or not (Bigger, 1944; Lennon and Jones, 2011; Orman and Brynildsen, 2013a; Wakamoto et al., 2013; Harms et al., 2016; Ueno et al., 2019). Thus, further characterization is needed to precisely define the persister metabolism. Of note, the majority of the work deals with persisters at the whole population level, while the persister cells are more likely stochastically generated due to individual phenotype switches (Balaban et al., 2019). It is therefore more appropriate and precise to explore their survival mechanisms and pathogenicity-related metabolism at a single-cell level (Balaban et al., 2019; Goormaghtigh and Van Melderen, 2019). As such, SCRS as a well-established and powerful tool has been widely used in biological and biomedical studies (Wang et al., 2020). Unfortunately, this non-destructive, label-free, and molecular-specific approach has rarely been applied for pioneering investigations on persister cells.

In the present study, SCRS-based Raman spectra of *E. coli* and its persisters from different phases are recorded and analyzed. According to previous studies, different peaks of the Raman spectra are related to specific chemical bonds of major cellular components and metabolites (De Gelder et al., 2007; Movasaghi et al., 2007). Our findings show that after ampicillin treatment at 100 μg/mL for 4 h, *E. coli* persister cells exhibit reduced nucleic acid-related bands when compared to their counterparts, suggesting the persisters may show a lower rate of cell replication. Interestingly, enhanced lipid-, polysaccharide-, and most protein-related bands, except for those related to amide, are observed within the persister cells with reference to *E. coli* cells, indicating that after 4 h of treatment with ampicillin, the persisters accumulate more cellular components than the *E. coli* cells. Combined with the result of nucleic acid, persisters could undertake higher synthesis activity, although their cell cycles are greatly arrested.

The PCA and t-SNE, the two common unsupervised tagging machine learning methods, have been successfully used to visually display the data structure. In addition, PLS-DA and SVM are employed for modeling the data from different experimental groups. Herein, *E. coli* persisters formed in different culture stages and their counterpart are then precisely classified into four spaces for their signature phenotypes on the basis of their Raman spectra. Thus, Raman spectroscopy could be a powerful tool to identify persisters and normal bacteria at the single-cell level. The present study provides the first report on SCRS-based identification of *E. coli* persisters and the notable advantages of this fast, non-invasive, and reliable approach. It is worthy to apply this favorable technique for investigating other microorganisms and their persisters.

Moreover, the D_2_O-Ramanometry has been used for quantifying the metabolic activities of *E. coli* and its persisters as previously described by our laboratory studies (Berry et al., 2015). Importantly, the CDR [CD/(CD + CH)] strength of *E. coli* is in line with the concentration and time of D_2_O treatment, highlighting that it could serve as a novel quantitative marker for single-cell metabolic activity. Interestingly, *E. coli* persisters exhibit higher metabolic activities than their counterparts, revealing that these persisters are exposed to adverse challenges, such as antibiotic treatment, that need augmented metabolism for survival even though they appear to show growth arrest.

In order to effectively tackle microbial persisters, it is of great importance to understand how they form and figure out how they resuscitate (Song and Wood, 2020a). Traditional approaches investigate the resuscitation of persister cells by detecting the changes in their turbidity in broth (Curry et al., 2021), recording colony formation units on an agar plate (Mohiuddin et al., 2020a; Song and Wood, 2020b; Yamasaki et al., 2020), observing the resuscitation using time-lapse microscopy (Sulaiman and Lam, 2020), and performing flow cytometry (Mohiuddin et al., 2020b). In the present study, we have explored the changes in cellular components within *E. coli* persisters after their resuscitation using SCRS. *E. coli* persisters largely reduce their replication rate after ampicillin treatment, as nucleic acid-related bands show a significant decrease. After 4-h resuscitation, a slight recovery in nucleic acid-related bands is observed in persisters, while the intensity remains to be lower than their counterparts. Moreover, amide exhibits a similar trend as nucleic acid, indicating that it might be involved in cell replication. In contrast, several other major cellular components and metabolites, such as lipid-, polysaccharide-, and most protein-related bands, remarkably increase in persister cells and partly recover after 4-h resuscitation as shown in Figure 6, indicating that *E. coli* persisters enhance their synthesis activity in response to the harsh challenges of antibiotic treatment. Taken together, SCRS as a new approach enables us to determine the relative replication rate and biochemical synthesis level of persister cells in a rapid and convenient manner.

Furthermore, the metabolic activities of *E. coli* persisters during resuscitation are assessed *via* D_2_O-Ramanometry, and the results demonstrate that persister cells exhibit lower metabolic activities than *E. coli* cells during and after the 4-h awaking period. Indeed, persister cells promptly absorb D_2_O within 1 h after the termination of antibiotic treatment, which is consistent with the finding of Mohiuddin and co-workers (Mohiuddin et al., 2020b). Additionally, persister cells maintain a constant and stable absorption rate during the short resuscitation stage. This observation is different from the untreated *E. coli* cells that show high metabolic activity in the first hour and low yet consistent activity for the following 3 h. It has actually been reported that persister cells are able to recover fully at the exponential state, once cell division resumes after resuscitation (Kim et al., 2018).

Generally, the gut microbiome consists of a complex and dynamic population of microorganisms, which strive to live in the face of the harsh challenges from other competitors, antimicrobials, and the host, yet shape the host to a large extent (Thursby and Juge, 2017). As one of the first colonizers in the gut after birth, *E. coli* critically accounts for a beneficial environment to facilitate the colonization of subsequent microorganisms and microbe–host symbiosis (Secher et al., 2016). On the other hand, some species of *E. coli* are crucially involved in various systemic diseases and disorders (Kaper et al., 2004). Over the past two decades, *E. coli* persisters have been intensively investigated (Balaban et al., 2004; Dorr et al., 2010; Orman and Brynildsen, 2013b; Shan et al., 2017; Sulaiman and Lam, 2020). Based on the advanced SCRS technology, our study has assessed for the first time the relative levels of biochemical synthesis in *E. coli* persister cells during their formation and resuscitation, indicating that these persister cells may develop intrinsic strategies *via* downregulating the replication rate while enhancing the biosynthesis for survival and subsequent recovery. Importantly, the findings from D_2_O-Ramanometry further reveal the metabolic activity of *E. coli* persisters under different conditions and an increased level of metabolism in *E. coli* persisters with reference to *E. coli* cells. Referring to previous studies, there are two important improvements in our investigation. First, the biochemical synthetic and metabolic activities of the *E. coli* persister cells were analyzed on the basis of their molecular quantity, instead of merely observing the dividing rate and/or growth state. Indeed, the present study has provided a deeper insight into the phenotype of these persisters. Second, our observations arise from appropriate investigations of every single cell rather than the “average” level of the whole bacterial population of bacterial persisters. Notably, this accurate approach could better reflect the characters of microbial persisters as randomly generated phenotypic variants (Balaban et al., 2019). Further investigations are needed to explore more differences between *E. coli* and *E. coli* persisters, and to explore more details regarding their metabolic activity. It is worthwhile to integrate and apply both SCRS and D_2_O-Ramanometry techniques for further studies on microbial persisters and related host responses.

Next, the transcriptome of *E. coli* persisters can be compared to those of the non-persister cells, on the basis of Raman-activated cell sorting and sequencing technologies (RACS-Seq) (He et al., 2019). We have recently demonstrated that such links of metabolic phenotype of targeting microbes and high-coverage genome sequences can be established at the resolution of the precisely single bacterial cell directly from human or soil microbiota (Xu et al., 2020; Jing et al., 2021; Liu et al., 2022), using Raman-activated gravity-driven single-cell encapsulation and sequencing (RAGE-Seq). Such efforts could promise to unveil the transcriptomic or epigenetic mechanisms that underpin microbial persistence in this model system.

There are some limitations of this study for elaboration. The *E. coli* persisters used in this study were produced according to a previously described method (Kaldalu et al., 2016). However, the remaining *E. coli* cells are not entirely pure persisters, but rather a mixture of cells with altered morphologies, transiently ampicillin-induced resistance/tolerance, and true persister cells. Thus, it could be appropriate to claim the remaining mixture as ampicillin-treated *E. coli* “persisters” that are actually the distinguishable subsets of *E. coli* cells that are highly refractory to the antibiotic treatment. Nevertheless, it is considered that *E. coli* persisters would make up the majority of them. In addition, it is highly challenging to directly detect their Raman spectra without ampicillin treatment, especially with the presence of non-persister cells as concerned above, partly due to the intrinsic limits of Raman spectroscopy (e.g., a requirement of a few seconds for testing every single cell).

## Conclusion

With the limitations of this study, our findings present the first evidence on the overall profiles of biochemical components in *E. coli* persisters using Raman spectroscopy, and quantification of their relative metabolic activities *via* the D_2_O-Ramanometry technique. SCRS and D_2_O-Ramanometry, as integrated powerful approaches, can be extended to investigate other microorganisms and their persisters. This advanced technology inspires us to further explore the signature profiles and underlying survival mechanisms of various microbial persisters in connection to the resultant immuno-inflammatory responses. Our current findings could contribute to establishing novel approaches to assessing microbial persisters and thereby developing refined tackling strategies for preventive healthcare and precision treatment.

## Data availability statement

The raw datasets generated in this study have been submitted to the database of Single-Cell Center, Qingdao Institute of BioEnergy and Bioprocess Technology, Chinese Academy of Sciences (http://mard.single-cell.cn/raw_spectrum_data/).

## Author contributions

LJ and JX designed the study. CW and RC carried out the experiments, analyzed the acquired data, and wrote the article. All authors reviewed the manuscript, contributed to the discussion, and approved the final version of the article.

## Funding

This study was supported by the Chinese Academy of Sciences (XDB29050400 and KFJ-STS-QYZX-087 to JX), the Hong Kong Research Grants Council (GRF/RGC No. 17119819 to LJ), and the Modern Dental Laboratory/HKU Endowment Fund to LJ.

## Conflict of interest

The authors declare that the research was conducted in the absence of any commercial or financial relationships that could be construed as a potential conflict of interest.

## Publisher's note

All claims expressed in this article are solely those of the authors and do not necessarily represent those of their affiliated organizations, or those of the publisher, the editors and the reviewers. Any product that may be evaluated in this article, or claim that may be made by its manufacturer, is not guaranteed or endorsed by the publisher.
