# Supplementary material for: Single-cell Raman spectroscopy identifies Escherichia coli persisters and reveals their enhanced metabolic activities
Source: Front Microbiol. 2022 Aug 4;13:936726. doi: 10.3389/fmicb.2022.936726 (PMC9386477; doi:10.3389/fmicb.2022.936726)
Supplement: Supplementary file 1 [file Data_Sheet_1.docx]

***Supplementary Material***

**Supporting Tables**

**TABLE S1** Raman bands related to major cellular components and metabolites

| Wavenumber (cm^−1^) | Molecular assignment | Components | References |
| --- | --- | --- | --- |
| 667 | Guanine, Thymine (ring breathing modes in the DNA bases), tyrosine-G backbone in RNA | Nucleic acids | (Maquelin et al., 2002; Boschetto et al., 2018) |
| 724 | Adenine | Nucleic acids | (Maquelin et al., 2002; Teng et al., 2016; Germond et al., 2018) |
| 780 | Cytosine, uracil | Nucleic acids | (Strola et al., 2014; Teng et al., 2016; Xuan Nguyen et al., 2017; Germond et al., 2018) |
| 806 | Nucleic acids (C-O-P-O-C in RNA backbone) | Nucleic acids | (Maquelin et al., 2000; Teng et al., 2016; Germond et al., 2018) |
| 850 | Polysaccharide structure | Polysaccharide | (Gniadecka et al., 1997; Gerbino et al., 2012) |
| 924 | C-C stretching of proline ring | Protein | (Stone et al., 2000; Movasaghi et al., 2007) |
| 1003 | Phenylalanine | Phenylalanine | (Maquelin et al., 2000; De Gelder et al., 2007; Strola et al., 2014; Germond et al., 2018) |
| 1044 | Proline | Protein | (Frank et al., 1995; De Gelder et al., 2007) |
| 1127 | ν(C-C) skeletal of acyl backbone in lipid (transconformation) | Lipid | (Maquelin et al., 2002; Cheng et al., 2005; Teng et al., 2016) |
| 1205 | Amide III and CH2 wagging vibrations from glycine backbone and proline side chains | Protein | (Dukor, 2001; Germond et al., 2018; Verma et al., 2021) |
| 1481 | Amide II | Amide | (Dukor, 2001; Movasaghi et al., 2007) |
| 1556 | Amide II | Amide | (Maquelin et al., 2000; Movasaghi et al., 2007; Kourkoumelis et al., 2018) |
| 1657 | Amide I | Amide | (Maquelin et al., 2000; Maquelin et al., 2002; Strola et al., 2014; Xuan Nguyen et al., 2017) |
| 2854 | Contributions from acyl chains | Lipid | (Krafft et al., 2005; Verma et al., 2021) |

**TABLE S2A** Data modeling by Partial Least Squares Discrimination Analysis (PLS-DA)

| **PLS-DA** | **3h *E. coli*^T^** | **3h persisters^T^** | **6h *E. coli*^T^** | **6h persisters^T^** | **Accuracy** |
| --- | --- | --- | --- | --- | --- |
| 3h *E. coli*^P^ | 48 | 0 | 2 | 0 | 96% |
| 3h persisters^P^ | 0 | 43 | 0 | 0 | 100% |
| 6h *E. coli*^P^ | 0 | 0 | 45 | 0 | 100% |
| 6h persisters^P^ | 0 | 0 | 0 | 41 | 100% |

**TABLE S2B** Data modeling by Support Vector Machine (SVM)

| **SVM** | **3h *E. coli*^T^** | **3h persisters^T^** | **6h *E. coli*^T^** | **6h persisters^T^** | **Accuracy** |
| --- | --- | --- | --- | --- | --- |
| 3h *E. coli*^P^ | 48 | 0 | 0 | 0 | 100% |
| 3h persisters^P^ | 0 | 43 | 0 | 0 | 100% |
| 6h *E. coli*^P^ | 0 | 0 | 47 | 0 | 100% |
| 6h persisters^P^ | 0 | 0 | 0 | 41 | 100% |

P: Prediction. T: True.

**Supporting Figures**


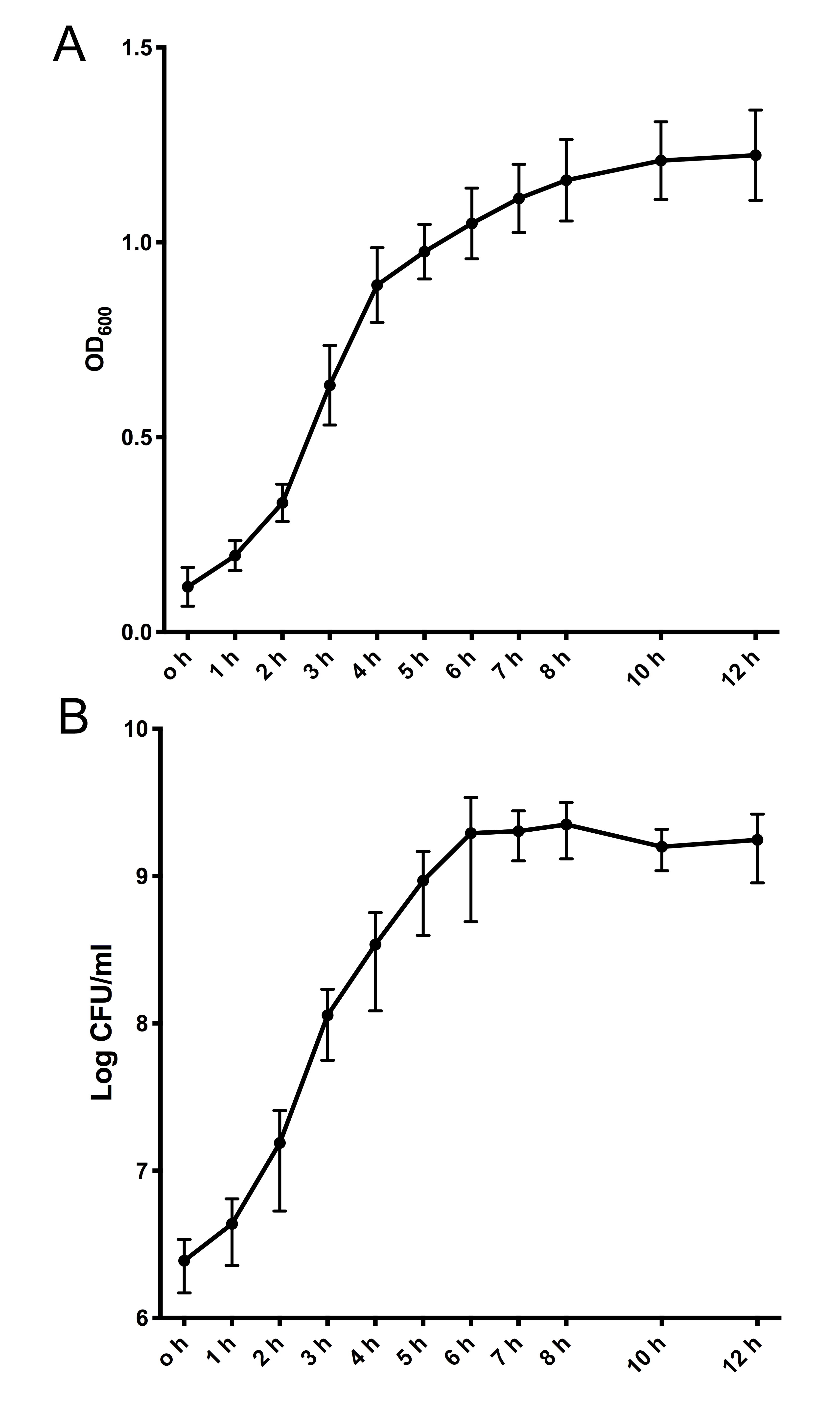


**Supplementary Figure 1. The** **growth curve of *E. coli*.** Overnight planktonic-cultured *E. coli* was 1:100 diluted into fresh LB broth and then cultured at 37°C with 200 rpm shaking. At different time points, aliquots were taken from the tube and OD_600_ was recorded **(A)**. At the same time, aliquots taken from the same tube were 10-fold serially diluted in PBS, followed by plate-culture to determine bacterial viability by CFU counts **(B)**.

**
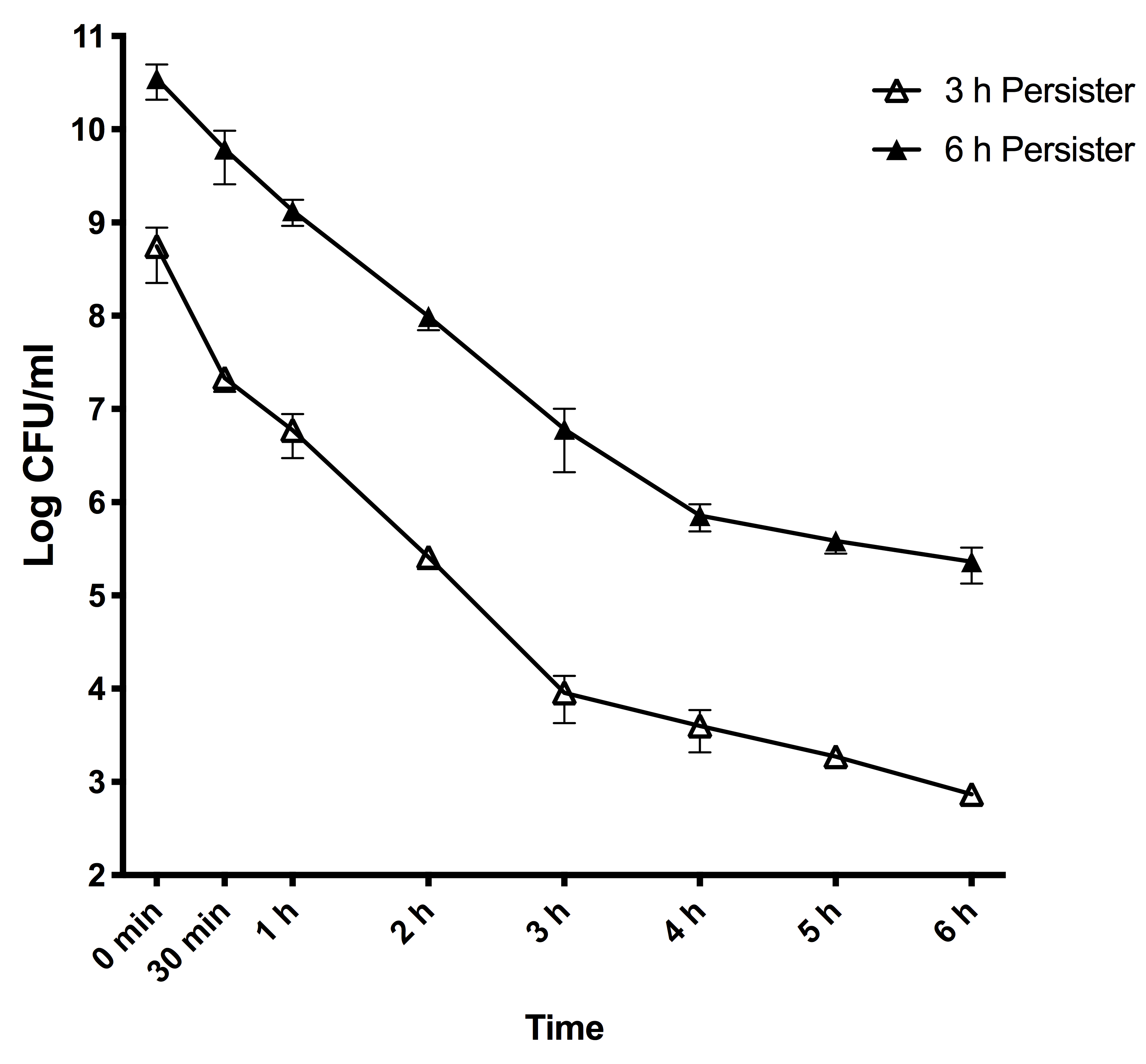
**

**Supplementary Figure 2. The biphasic kill curve of *E. coli* persisters.** Overnight planktonic-cultured *E. coli* (12 h) was 1:100 diluted into fresh LB broth and further cultured for 3 h and 6 h, followed by ampicillin treatment (100 μg/mL, 32×MIC) for 4 h. Aliquots were washed twice with PBS and 10-fold serially diluted in PBS, followed by plate-culture to test bacterial viability by CFU counts.

**
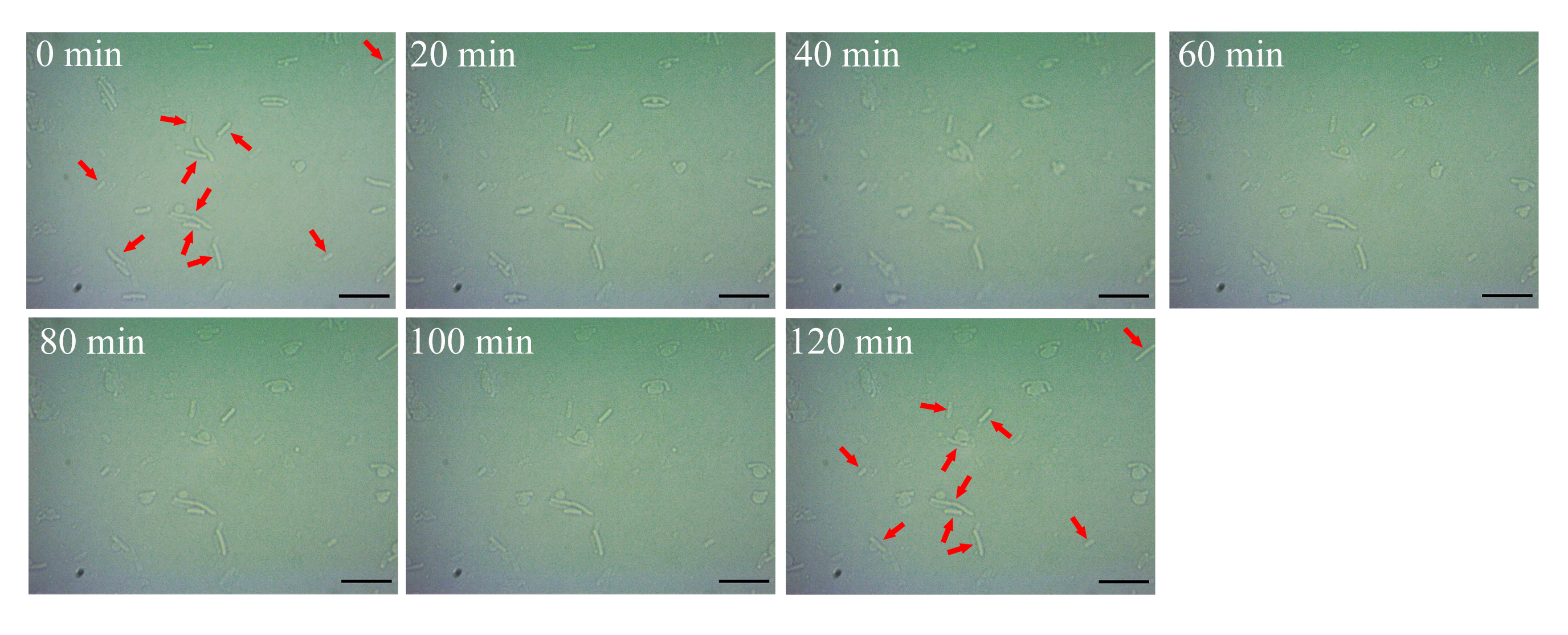
**

**Supplementary Figure 3. Non-growing of *E. coli* persisters.** Overnight planktonic-cultured *E. coli* (12 h) was 1:100 diluted into fresh LB broth and further cultured for 3 h, followed by ampicillin treatment (100 μg/mL, 32×MIC) for 4 h. Persister cells were re-suspended with fresh LB with ampicillin at 100 μg/mL, and the cell growth was observed and recorded using agarose pad containing ampicillin (100 μg/mL) as described in the main text. Red arrow points to the persister cells with neither growth nor lysis during the 120 min observed. Scale bar: 5 μM.

**
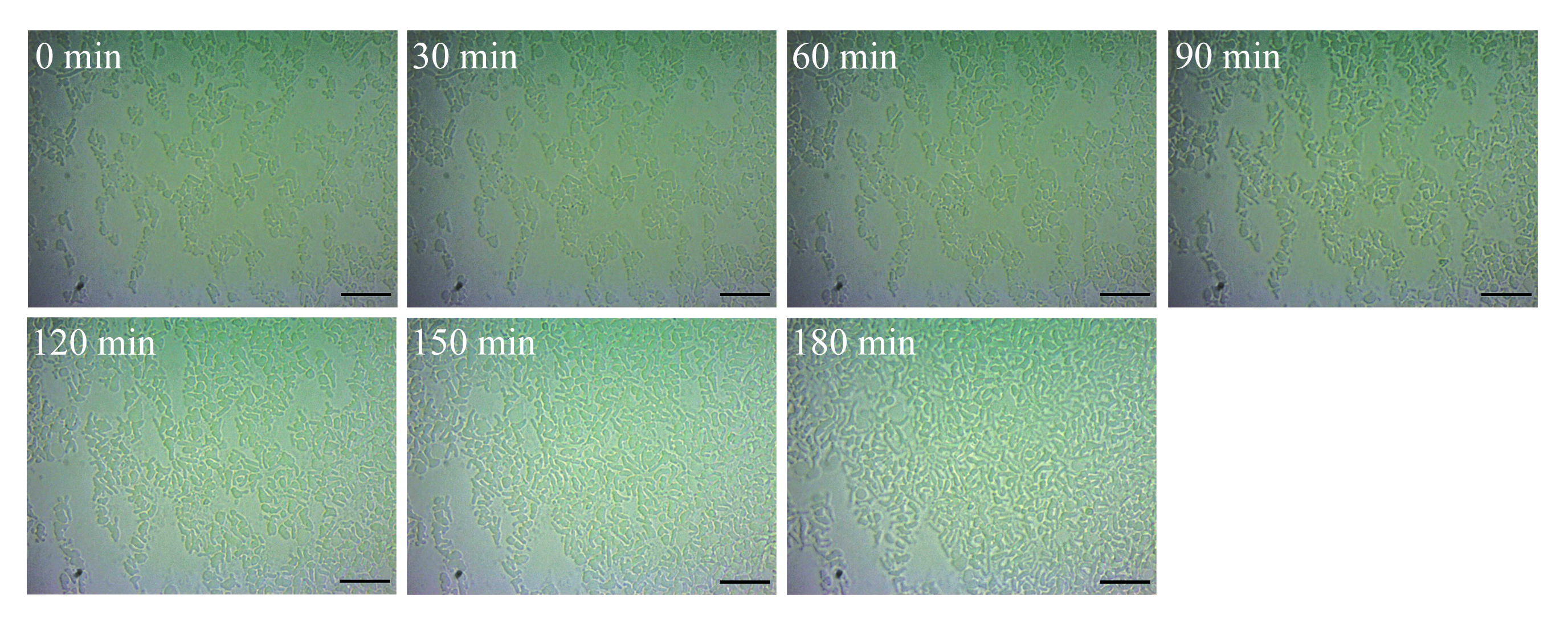
**

**Supplementary Figure 4. Resuscitation of *E. coli* persisters after termination of antibiotic treatment.** Overnight planktonic-cultured *E. coli* (12 h) was 1:100 diluted into fresh LB broth and further cultured for 3 h, followed by ampicillin treatment (100 μg/mL, 32×MIC) for 4 h. Persister cells were washed twice with PBS and re-suspended with fresh LB without ampicillin. Cell growth was observed and recorded using agarose pad (without ampicillin) as described in the main text. Scale bar: 5 μM.


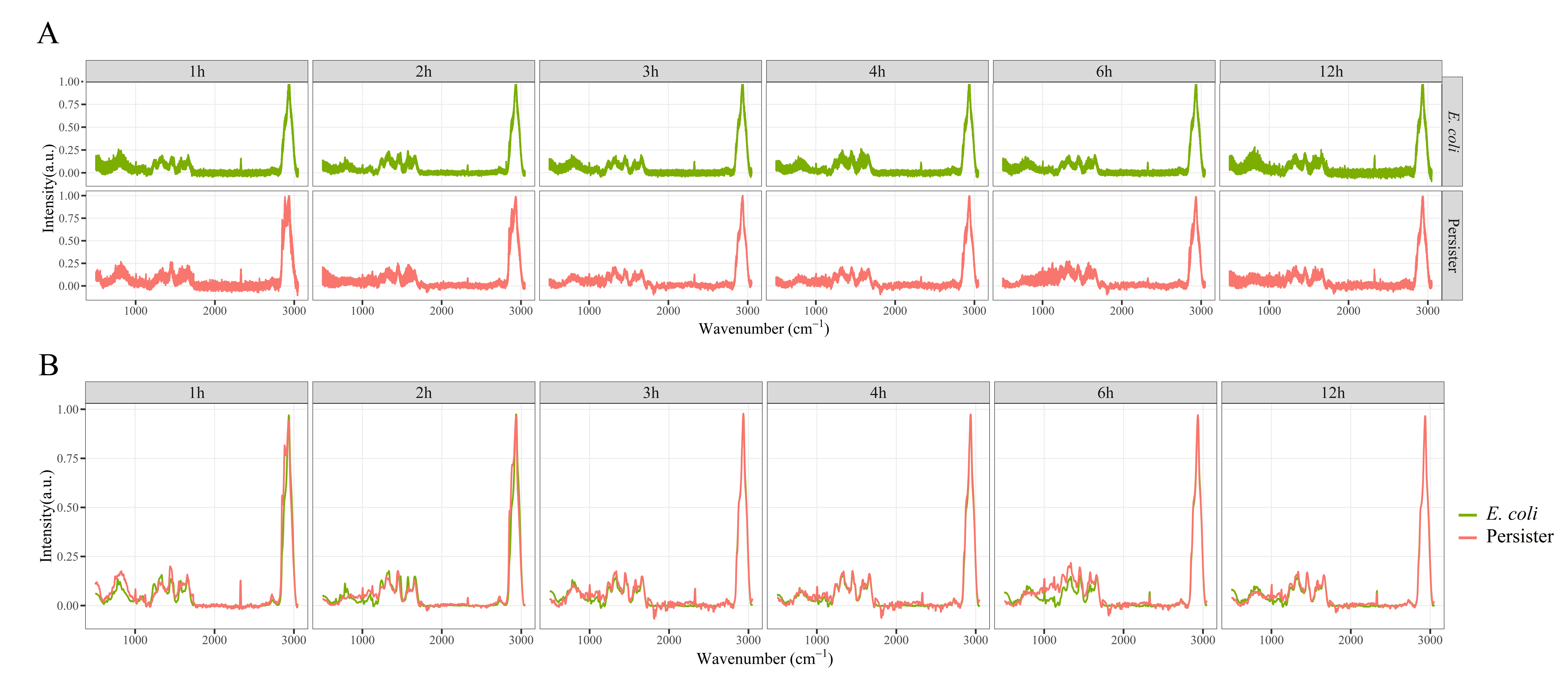


**Supplementary Figure 5. Single-cell Raman spectra of *E. coli* and its persister cells.** Overnight planktonic-cultured *E. coli* (12 h) was 1:100 diluted into fresh LB broth and further cultured for 3 h and 6 h, followed by ampicillin treatment (100 μg/mL, 32×MIC) for 4 h. Single-cell Raman spectra of *E. coli* and its persister cells were recorded at designed time points (1 h, 2 h, 3 h, 4 h, 6 h and 12 h). Raman spectra of each single cell **(A)** and average **(B)**.

**
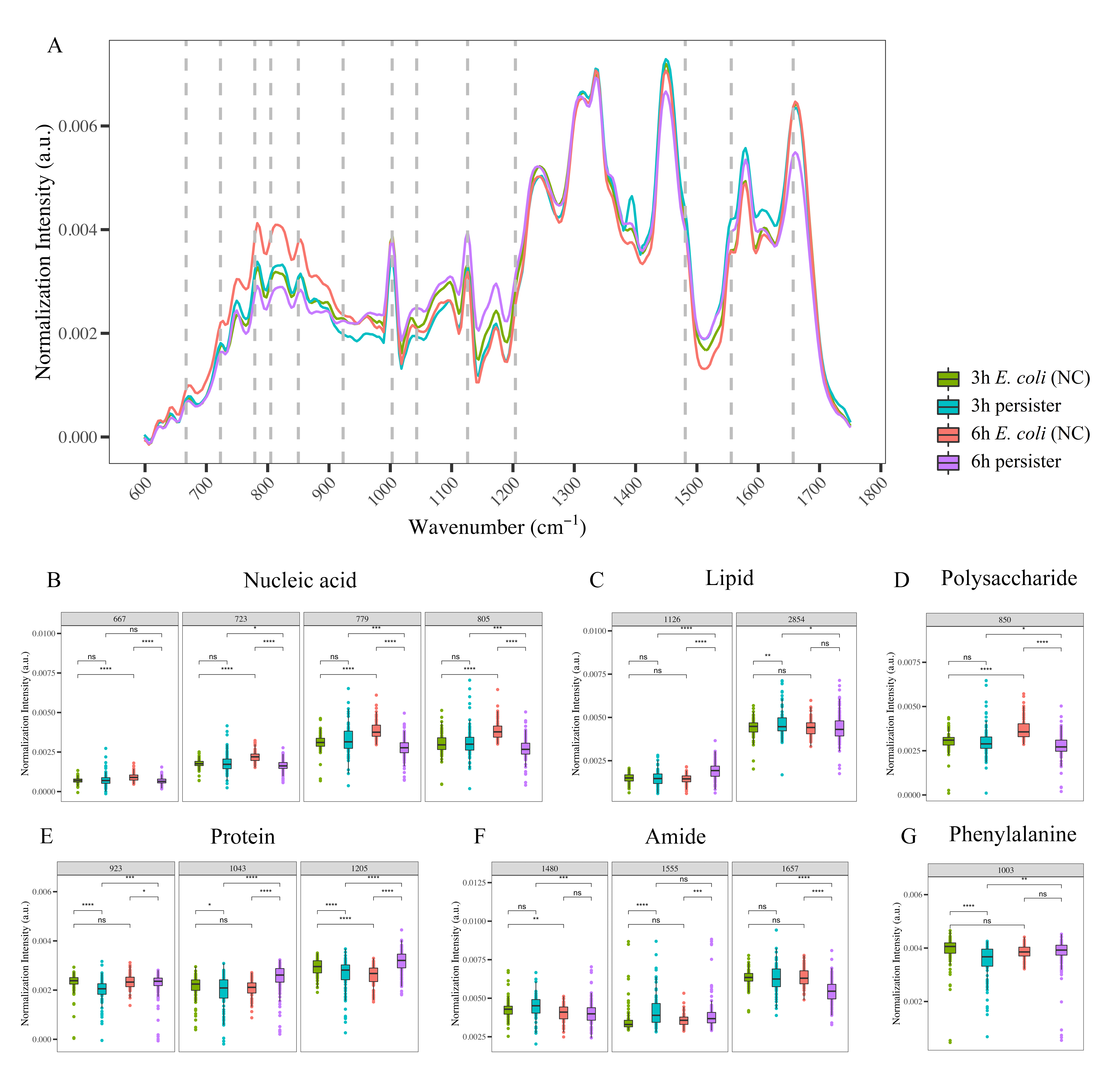
**

**Supplementary Figure 6. Single-cell Raman spectra of *E. coli* persisters after 24-h antibiotic treatment.** Overnight planktonic-cultured *E. coli* (12 h) was 1:100 diluted into fresh LB broth and further cultured for 3 h and 6 h, followed by ampicillin treatment (100 μg/mL, 32×MIC) for 24 h. Single-cell Raman spectra were acquired afterwards. **(A)** Mean spectra of *E. coli* and persisters, normalized by the sum of fingerprint area from 600 cm^-1^ to 1750 cm^-1^. **(B to G)** Intensity of Raman bands for major cellular components: nucleic acid **(B)**, lipid **(C)**, polysaccharide **(D)**, proteins **(E)**, amide **(F)** and phenylalanine **(G)**. ns: no significance; NC: *E. coli* cells without ampicillin treatment; * *P* < 0.05; ** *P* < 0.01; *** *P* < 0.001, **** *P* < 0.0001.

**
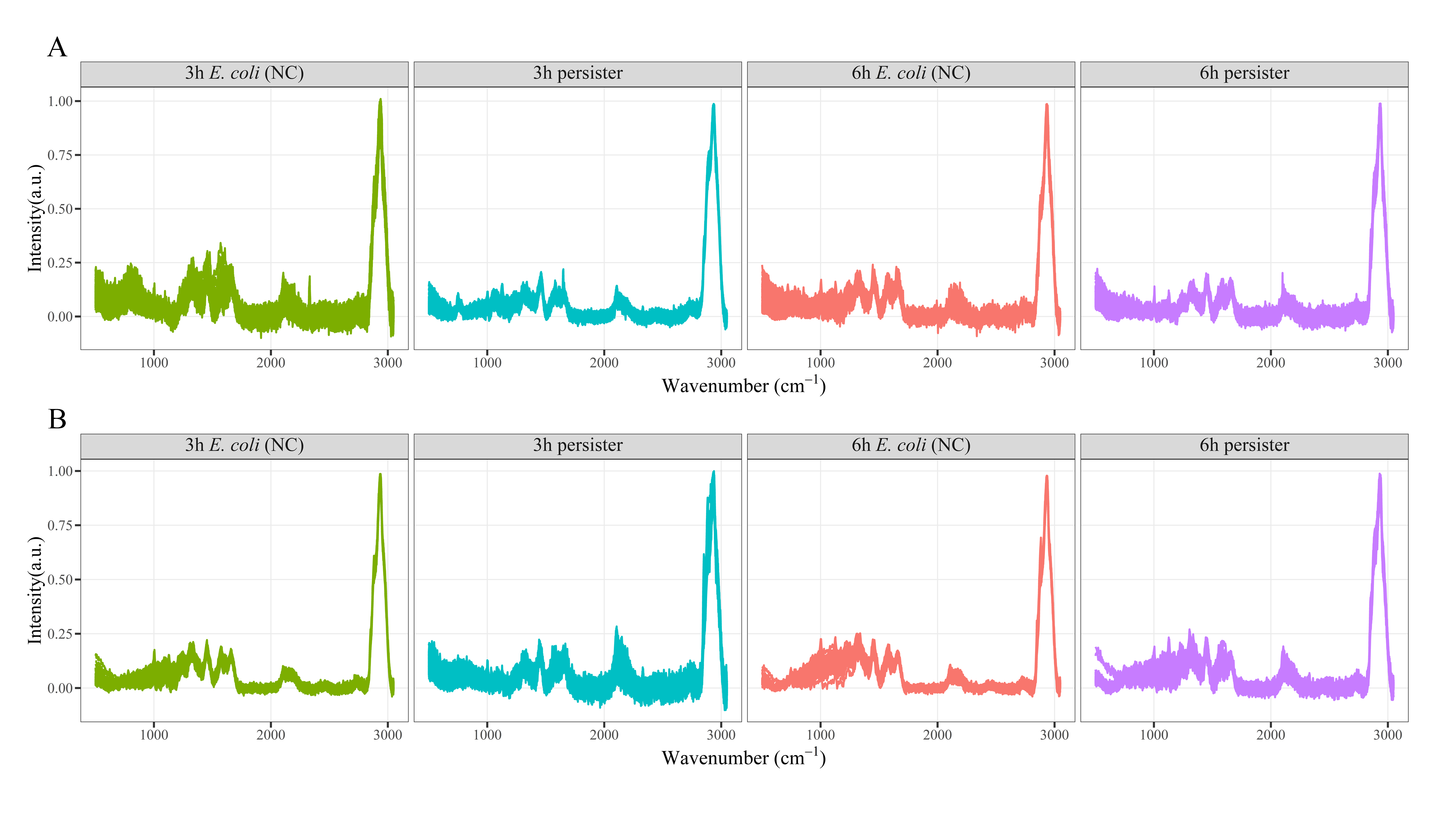
**

**Supplementary Figure 7. Single-cell Raman spectra of *E. coli* and its persister cells labeled by D_2_O.** D_2_O was used to detect the metabolic activities of *E. coli* and its persisters. **(A)**: *E. coli* cells at 3 h and 6 h were cultured in LB broth with 100% of D_2_O and ampicillin at 100 μg/mL for 4 h. **(B)**: *E. coli* cell in 3 h and 6 h were treated with ampicillin (100 μg/mL, 4 h) for persister formation, followed by culture in LB broth with 100% of D_2_O for another 4 h. Single-cell Raman spectra were acquired after D_2_O labelling.


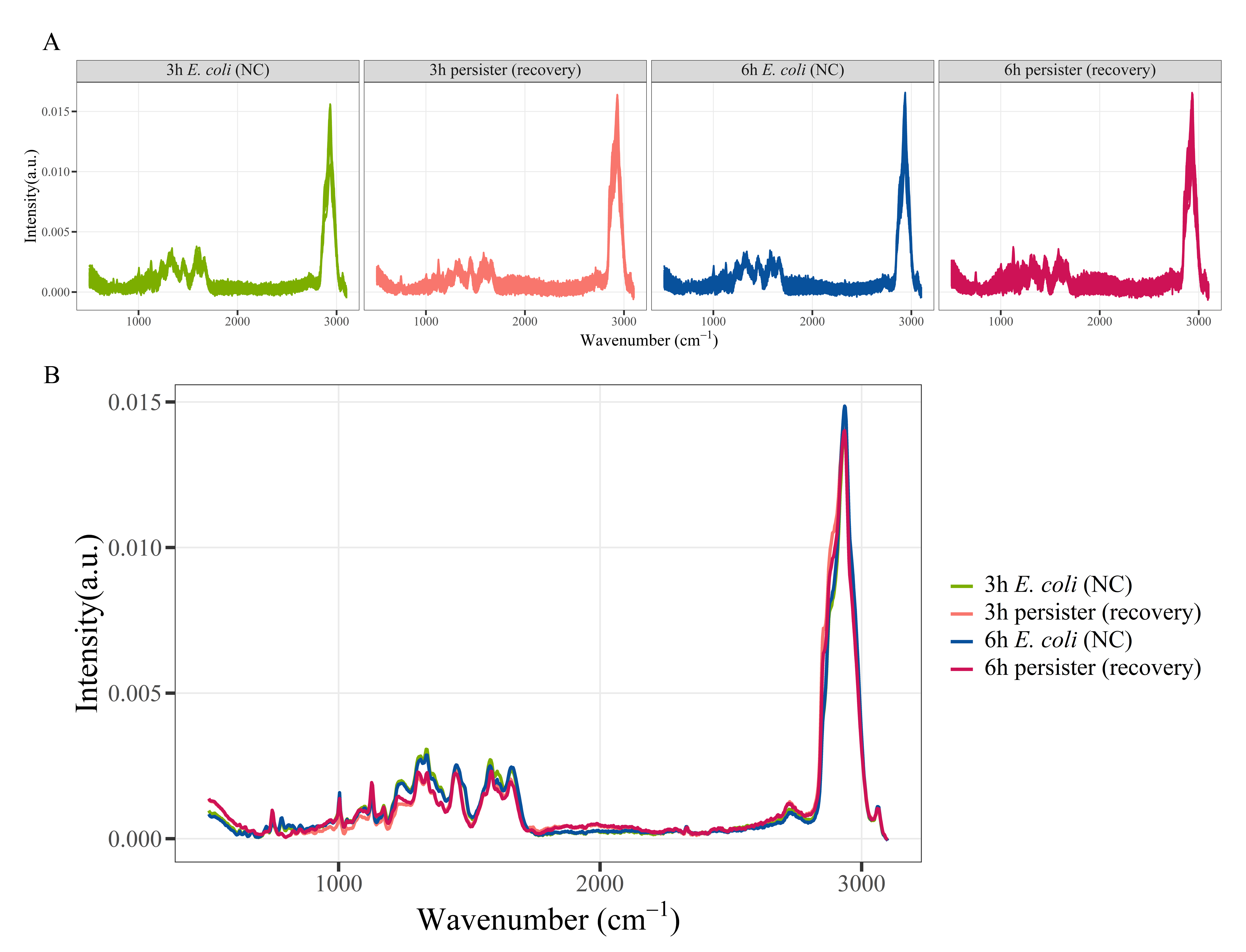


**Supplementary Figure 8.** **Single-cell Raman spectra of *E. coli* and its persister cells after 4h-resuscitation.** Overnight planktonic-cultured *E. coli* (12 h) was 1:100 diluted into fresh LB broth and further cultured for 3 h and 6 h, followed by ampicillin treatment (100 μg/mL, 32×MIC) for 4 h. Then, the persisters were washed twice with Milli-Q water and further cultured in fresh LB broth without drug for another 4 h. Single-cell Raman spectra were acquired afterwards. Raman spectra of each single cell **(A)** and average **(B)**.

**
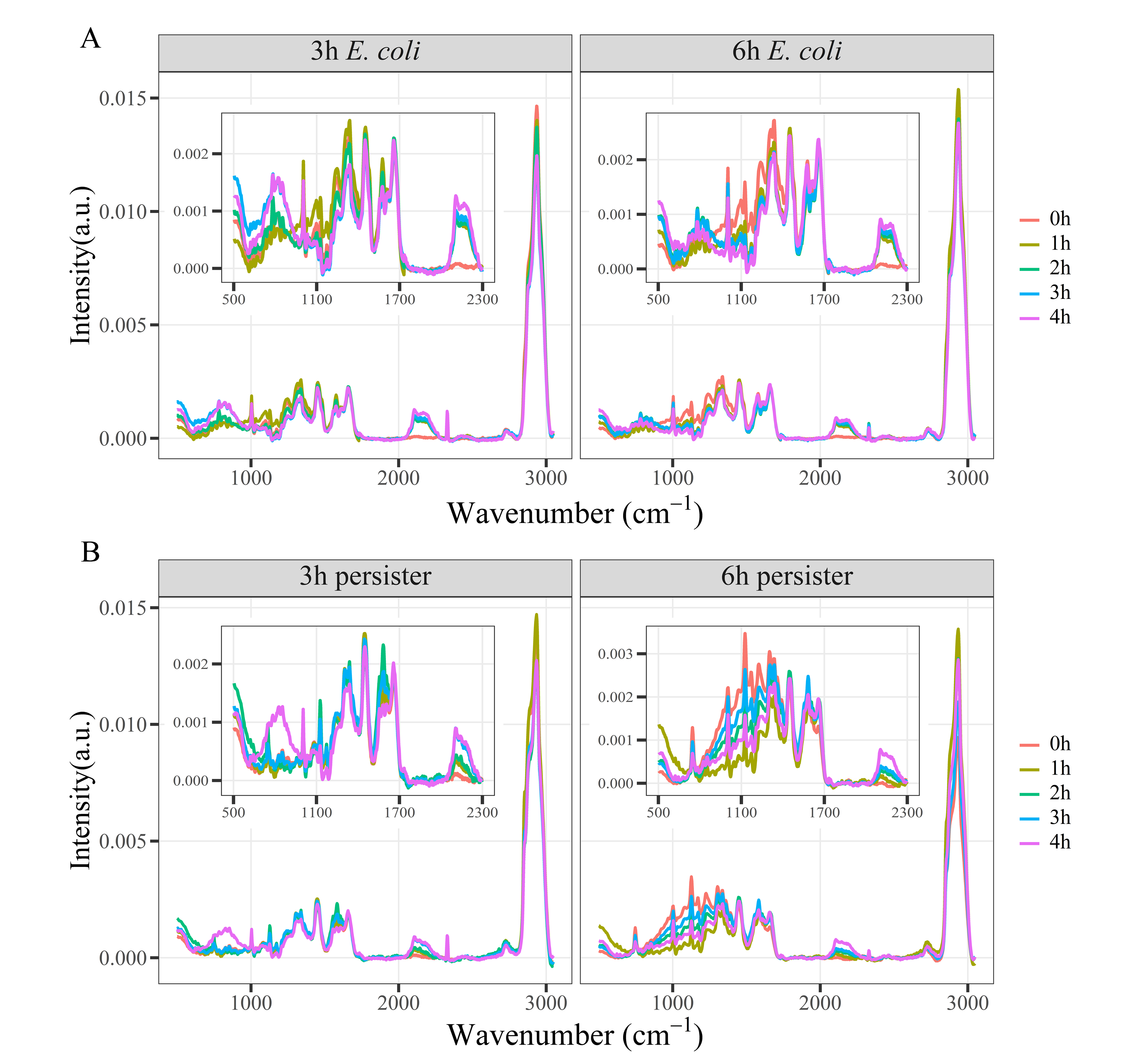
**

**Supplementary Figure 9. Average Single-cell Raman spectra of *E. coli* and its persister cells during the resuscitation.** Overnight planktonic-cultured *E. coli* (12 h) was 1:100 diluted into fresh LB broth and further cultured for 3 h and 6 h, followed by ampicillin treatment (100 μg/mL, 32×MIC) for 4 h. Then, the persisters were washed twice with Milli-Q water and further cultured in fresh LB broth without drug for another 4 h. Single-cell Raman spectra of *E. coli* **(A)** and its persisters **(B)** were acquired at different time points.

**References**

Boschetto, F., Adachi, T., Horiguchi, S., Fainozzi, D., Parmigiani, F., Marin, E., et al. (2018). Monitoring metabolic reactions in Staphylococcus epidermidis exposed to silicon nitride using in situ time-lapse Raman spectroscopy. *J Biomed Opt* 23**,** 1-10. doi: 10.1117/1.Jbo.23.5.056002

Cheng, W.T., Liu, M.T., Liu, H.N., and Lin, S.Y. (2005). Micro-Raman spectroscopy used to identify and grade human skin pilomatrixoma. *Microsc Res Tech* 68**,** 75-79. doi: 10.1002/jemt.20229

De Gelder, J., De Gussem, K., Vandenabeele, P., and Moens, L. (2007). Reference database of Raman spectra of biological molecules. *J Raman Spectrosc* 38**,** 1133-1147. doi: 10.1002/jrs.1734

Dukor, R.K. (2001). "Vibrational Spectroscopy in the Detection of Cancer," in *Handbook of Vibrational Spectroscopy*.).

Frank, C.J., Mccreery, R.L., and Redd, D.C. (1995). Raman spectroscopy of normal and diseased human breast tissues. *Anal Chem* 67**,** 777-783. doi: 10.1021/ac00101a001

Gerbino, E., Mobili, P., Tymczyszyn, E.E., Frausto-Reyes, C., Araujo-Andrade, C., and Gómez-Zavaglia, A. (2012). Use of Raman spectroscopy and chemometrics for the quantification of metal ions attached to *Lactobacillus kefir*. *J Appl Microbiol* 112**,** 363-371. doi: 10.1111/j.1365-2672.2011.05210.x

Germond, A., Ichimura, T., Horinouchi, T., Fujita, H., Furusawa, C., and Watanabe, T.M. (2018). Raman spectral signature reflects transcriptomic features of antibiotic resistance in *Escherichia coli*. *Commun Biol* 1**,** 85. doi: 10.1038/s42003-018-0093-8

Gniadecka, M., Wulf, H.C., Nymark Mortensen, N., Faurskov Nielsen, O., and Christensen, D.H. (1997). Diagnosis of Basal Cell Carcinoma by Raman Spectroscopy. *J Raman Spectrosc* 28**,** 125-129. doi: 10.1002/(sici)1097-4555(199702)28:2/3<125::Aid-jrs65>3.0.Co;2-#

Kourkoumelis, N., Gaitanis, G., Velegraki, A., and Bassukas, I.D. (2018). Nail Raman spectroscopy: A promising method for the diagnosis of onychomycosis. An ex vivo pilot study. *Med Mycol* 56**,** 551-558. doi: 10.1093/mmy/myx078

Krafft, C., Neudert, L., Simat, T., and Salzer, R. (2005). Near infrared Raman spectra of human brain lipids. *Spectrochimica acta. Part A, Molecular and biomolecular spectroscopy* 61**,** 1529-1535. doi: 10.1016/j.saa.2004.11.017

Maquelin, K., Choo-Smith, L.P., Van Vreeswijk, T., Endtz, H.P., Smith, B., Bennett, R., et al. (2000). Raman spectroscopic method for identification of clinically relevant microorganisms growing on solid culture medium. *Anal Chem* 72**,** 12-19. doi: 10.1021/ac991011h

Maquelin, K., Kirschner, C., Choo-Smith, L.P., Van Den Braak, N., Endtz, H.P., Naumann, D., et al. (2002). Identification of medically relevant microorganisms by vibrational spectroscopy. *Journal of microbiological methods* 51**,** 255-271. doi: 10.1016/s0167-7012(02)00127-6

Movasaghi, Z., Rehman, S., and Rehman, I.U. (2007). Raman Spectroscopy of Biological Tissues. *Appl Spectrosc Rev* 42**,** 493-541. doi: 10.1080/05704920701551530

Stone, N., Stavroulaki, P., Kendall, C., Birchall, M., and Barr, H. (2000). Raman spectroscopy for early detection of laryngeal malignancy: preliminary results. *Laryngoscope* 110**,** 1756-1763. doi: 10.1097/00005537-200010000-00037

Strola, S.A., Baritaux, J.C., Schultz, E., Simon, A.C., Allier, C., Espagnon, I., et al. (2014). Single bacteria identification by Raman spectroscopy. *J Biomed Opt* 19**,** 111610. doi: 10.1117/1.JBO.19.11.111610

Teng, L., Wang, X., Wang, X., Gou, H., Ren, L., Wang, T., et al. (2016). Label-free, rapid and quantitative phenotyping of stress response in *E. coli* via ramanome. *Sci Rep* 6**,** 34359. doi: 10.1038/srep34359

Verma, T., Annappa, H., Singh, S., Umapathy, S., and Nandi, D. (2021). Profiling antibiotic resistance in *Escherichia coli* strains displaying differential antibiotic susceptibilities using Raman spectroscopy. *J Biophotonics* 14**,** e202000231. doi: 10.1002/jbio.202000231

Xuan Nguyen, N.T., Sarter, S., Hai Nguyen, N., and Daniel, P. (2017). Detection of molecular changes induced by antibiotics in *Escherichia coli* using vibrational spectroscopy. *Spectrochimica acta. Part A, Molecular and biomolecular spectroscopy* 183**,** 395-401. doi: 10.1016/j.saa.2017.04.077
